# Sex and gender effects on incidence of migraine and stroke: a longitudinal observational study based on the german socio-economic panel

**DOI:** 10.1186/s13293-026-00875-z

**Published:** 2026-03-16

**Authors:** Mascha Kern, Hans-Aloys Wischmann, Alex Müller, Tobias Kurth, Stefanie Theuring

**Affiliations:** 1 Institute of Public Health, Charité – Universitätsmedizin, Berlin, Corporate Member of Freie Universität Berlin and Humboldt-Universität zu Berlin, Berlin, Germany; 2 Institute of International Health, Charité Center for Global Health, Charité – Universitätsmedizin, Berlin, Corporate Member of Freie Universität Berlin and Humboldt-Universität zu Berlin, Berlin, Germany; 3 Gender Health and Justice Research Unit, University of Cape Town, Cape Town, South Africa

## Abstract

**Background:**

Sex and gender both affect health outcomes, often in complex ways that intertwine biological and social influences. While researchers have criticized the conflation of sex and gender in quantitative studies, it remains a challenge to analytically disentangle them. We investigated an approach to conceptualize sex and gender as interrelated constructs embedded within a specific social context and estimate their direct effects on health outcomes simultaneously.

**Methods:**

To analyze migraine and stroke incidence from 2011 until 2022, we used longitudinal data from the representative German Socio-Economic Panel (SOEP) within a causal framework. We applied a directed acyclic graph (DAG) to formalize hypothesized effects of sex and gender. Gender was modeled as an unobserved latent variable, influenced by sex assigned at birth and indicated by a set of gender-related variables. We codified the causal model as a structural equation model (SEM), enabling the joint estimation of the latent gender construct and the direct effects of both sex and gender on the health outcomes. We tested prior hypotheses in the SEM and explored additional relationships using causal discovery techniques.

**Results:**

Migraine incidence was 3.1 times higher among participants of female sex (6.2% vs. 2.0%). Sex had the strongest effect on reporting a new migraine diagnosis (*β* = 0.044, 95% CI [0.033, 0.056], *p* < .001), while gender showed no direct effect. Stroke incidence was lower among female respondents (1.2% vs. 1.7%), with sex showing a statistically significant negative effect, indicating that slightly fewer female persons reported a new stroke diagnosis (*β* = -0.010, 95% CI [-0.017, -0.004], *p* < .01). In contrast, gender showed a small, but significant positive effect (*β* = 0.006, 95% CI [0.002, 0.009], *p* < .01), suggesting that gender-related characteristics that were more frequently reported by female individuals had an effect on stroke incidence.

**Conclusions:**

Both sex and gender differentially affected stroke, whereas only sex showed a direct effect on migraine. We demonstrated that biological and social dimensions of sex and gender can be systematically addressed within the same model including gender as an unobserved latent variable, while remaining attentive to contextual complexity.

**Supplementary Information:**

The online version contains supplementary material available at 10.1186/s13293-026-00875-z.

## Abstract (plain language summary)

Sex and gender both influence health, but it is difficult to say which one has which effect. Sex is linked to biological factors, and gender is linked to social context. When people do quantitative research, they often either analyze them as if they were the same, or they only analyze one without the other. In our study, we explored whether it is possible to look at both sex and gender at the same time. We also analyzed how sex and gender together are related to health.

We used data from a large representative survey in Germany for the period between 2011 and 2022. We looked at how sex and gender influence migraine and stroke. We used a so-called causal diagram to show what our assumptions are, and did statistical tests in a so-called structural equation model (SEM). Since gender involves many different parts of a person’s social experience, we used a number of related factors to estimate it. We also assumed that gender was influenced by the sex a person was assigned at birth.

We found that participants with female sex developed migraine more often. This was only linked to their sex. Participants with female sex less often had a stroke – this was due to their sex. At the same time, we also saw that the risk of having a stroke was higher when participants of both sexes reported some of the gender-related factors mostly reported by female persons.

We showed that sex and gender influence stroke in different ways. Through our statistical analysis, we also showed how causal models can help study sex and gender at the same time, but as two separate factors. This shows how social and biological dimensions work together.

**Supplementary Information:**

The online version contains supplementary material available at 10.1186/s13293-026-00875-z.

## Highlights

We modeled sex and gender as distinct but interrelated constructs using a causal framework based on longitudinal data from the German Socio-Economic Panel.

Gender was operationalized as an unobserved latent variable, influenced by sex assigned at birth and indicated by multiple gender-related variables.

A structural equation model enabled simultaneous estimation of direct effects of sex and gender on migraine and stroke incidence.

Migraine incidence was significantly higher among participants of female sex and the effect was driven primarily by sex, while gender showed no direct effect.

Stroke incidence showed a distinct pattern: female sex had a protective effect, while gender-related characteristics commonly reported by female individuals were associated with a small but significant increase in stroke incidence.

**Supplementary Information:**

The online version contains supplementary material available at 10.1186/s13293-026-00875-z.

## Introduction

Sex and gender differences in neurological disorders regarding incidence, prevalence, prognosis, and risk factors are well established. The occurrence of migraine and stroke varies clearly between the sexes, and signs and symptoms are reported or present differently depending on gender, leading to divergent diagnostic patterns that may influence disease management [1–3].

Although awareness is growing that sex and gender refer to different concepts and are both relevant determinants of health, studies systematically including both sex and gender in design, analysis, and interpretation as a main consideration remain rare. Quantitative health research more frequently addresses the role of sex as a biological construct [2], encompassing sex assigned at birth, reproductive organs, sex chromosomes, and hormones [4]. The role of gender aspects – such as socially constructed norms, roles, relationships, social position, and power dynamics, grounded in structural societal inequities influencing behavior – often remain underexplored. Frequently, health researchers do not clearly operationalize sex and gender, conflating the two concepts [2, 5, 6], which can lead to a lack of clarity in research objectives and to misinterpretations of results. A more precise understanding of sex and gender holds the potential to make health research more equitable and representative, but also more scientifically accurate [5, 7].

Possible reasons for the lack of differentiation between sex and gender are the difficulty to assess gender in quantitative analyses and the lack of detailed information on gender-related components in already existing datasets [8–10]. It is possible to obtain measurements of sex through biological markers such as hormones or chromosomes, or, even if less accurate, by asking study participants to indicate their sex assigned at birth, or by checking the entry in birth registries. Gender can be more challenging to operationalize. While participants in inclusively designed studies can indicate their gender identity by specifying whether they define themselves as women, men, or non-binary individuals, other gender dimensions relevant to specific research questions may need to be considered [11, 12].

Recent approaches define gender as a multidimensional concept encompassing four dimensions: gender identity, gender roles and norms, gender relations, and institutionalized gender including societal power dynamics [11–14].

In recent years, scores and indices have been developed to measure the latent aspects of gender [15] as a dimensional variable rather than categorical using observed variables [16]. The most commonly used gender score method was first proposed in 1990 by Lippa and Connelly to assess differences in gender-related behaviors both within and across the sexes [17]. This method uses the probability of sex assigned at birth based on some gender-related variables as a measure of gender [17]. Gender is expected to influence people’s behavior and personal characteristics within their societal context and relations, resulting in gendered characteristics that are referred to as gender-related variables [18].

The gender score method has been controversially discussed [2, 13, 19], one main point of criticism being that the method suggests an ability to completely disentangle sex and gender [2, 13, 15]. It follows a two-step approach in which gender is first estimated as a composite score that is secondly applied in another model to estimate its effect on health differently from sex [18, 20]. Several researchers have noted that sex and gender cannot be easily divided into a dichotomy of biological vs. social [2, 21]. Both concepts interact with each other and with other social categories – such as racial or ethnic identity, immigration history, age, socioeconomic status, and sexual identity – to shape health [2, 22].

In this study, we applied an explicit causal framework and a life-course perspective to conceptualize potential effects of sex assigned at birth and gender as a latent construct on the incidence of migraine and stroke. We used the causal model to specify a structural equation model (SEM) that estimates a continuous gender variable based on observed variables from the population-representative German Socio-Economic Panel data (SOEP) [23]. Within the same SEM, we estimated direct effects of both sex and gender on the self-reported incidence of migraine and stroke diagnoses longitudinally from 2011 until 2022 in the German panel.

## Methods

### Conceptual framework and causal model

**Fig. 1:**
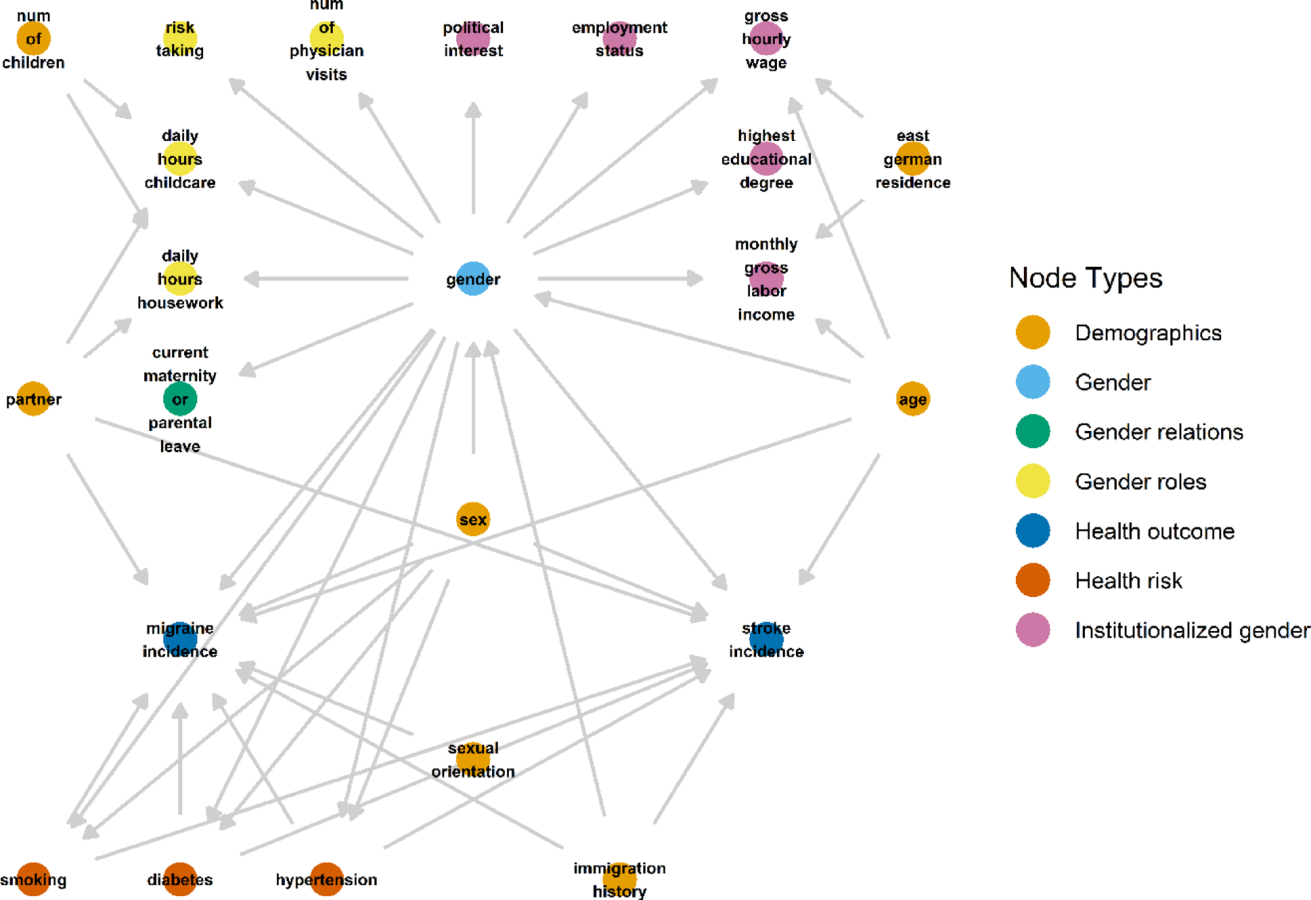
Directed acyclic graph (DAG) illustrating latent gender as a multidimensional construct, with its indicators organized by gender dimension. The graph depicts hypothesized relationships between latent gender, demographic variables, health risk factors, and health outcomes

We rely on a causal model to conceptualize gender as a latent variable based on observed gender-related variables as depicted in Fig. 1 that presents all selected variables and their assumed causal relationships, as elaborated on in the Metrics section, in a directed acyclic graph (DAG). DAGs are useful to capture a priori domain knowledge and codify assumptions about complex social structures as shown in previous sex/gender-research [15].

In a DAG, variables are depicted as “nodes”, while single-headed arrows indicate direct causal relationships between variables [24, 25]. A node that lies on a causal path between two nodes (e.g., A and B), represents an intermediate variable, which mediates the causal effect of the variable A on the variable B. Nodes that have an arrow pointing into node A, are called “parents of A”, and represent the set of variables that have a direct effect on the variable A [24, 25]. Arrows are included if the relationship is assumed to exist even only for some individuals, and the omission of an arrow, assuming no effect between nodes, is a stronger assumption than its inclusion [26, 27]. Variables that are assumed not to be caused by any other unmeasured or measured variable in the system are called “exogenous” [28]. The acyclic nature of DAGs ensures the absence of cycles; this implies that a variable cannot directly or indirectly cause itself [25, 27].

In our example, we consider sex assigned at birth as an exogenous variable. Gender, on the other hand, is the result of socialization processes that depend (among other factors) on a person’s sex assigned at birth. For example, if the sex of newborn babies is assigned female, they will be more likely to be socialized as girls [15]. For this reason, we consider ‘sex’ as a cause of ‘gender’ in the DAG in Fig. 1. This is, however, not a deterministic relationship, as gender is also shaped by other social and individual factors [15]. We added some of these assumed factors in the DAG: ‘Age’ was included as an exogenous variable accounting for differences in gender-related aspects (e.g., socio-temporal ideas about gender norms) [29] or generational changes. Further, to take migration-related factors into account that might shape gender due to socialization within different socio-cultural environments [30], we included the exogenous variable ‘immigration history’. ‘Age’ and ‘immigration history’ were considered effect measure modifiers for the effect of sex on gender [31], and an additional analysis was performed, stratified by age group.

Gender was modeled as an unmeasured latent variable that can only be inferred from other observed variables related to gender. We selected these variables based on previous research and according to three of the four gender dimensions described by Tannenbaum et al. – gender relations, gender roles, and institutionalized gender [11, 12]. We have classified the variables according to our understanding of these categories, but we are aware that a different classification would be possible depending on their definition [11, 32]. Based on this approach, we conceptualized gender as an individual characteristic referring to how a person performs gendered social characteristics within the norms of their social environment. This gender performance is viewed as socially constructed by systemic processes [21].

As shown in Fig. 1, latent gender exerts an influence on the selected gender-related variables, indicated by arrows pointing from gender to these variables. Given that certain gender-related variables may further be influenced by caregiving responsibilities, we specified regression models in which daily housework and childcare were treated as outcomes of the number of children in the household and the presence or absence of a partner. Similarly, we added regressions for the gross monthly labor income and the gross hourly wage as functions of age and residence in East German states.

Sex and gender aspects can both concurrently impact the health-related outcomes ‘incidence of migraine’ and ‘incidence of stroke’ via pathways related to sex hormones, allostatic load, chronic stress, and lifestyle factors [1, 3, 21]. We therefore drew arrows from both exposures to both outcomes. We acknowledge that knowing someone’s sex assigned at birth does not give us information about their hormone levels, chromosomes, or physiology [33], but since this information is rarely known and included in population-based research, we use sex assigned at birth as a proxy and acknowledge that this may lead to bias. Although some of the gender-related variables may influence the outcomes directly, we did not include these arrows, but hypothesized that the latent variable gender should account for the influences of its indicators.

To be consistent, we considered only the scenario when smoking, hypertension, and diabetes – that may be sex and/or gender-related [1, 3, 34] – were self-reported before the first diagnosis of migraine or stroke and added them as potential intermediates for the sex and gender effects on the health outcomes.

Finally, we added arrows from the variables ‘immigration history’ and ‘age’ to the health outcomes, as being a migrant can have an impact on healthcare access and being diagnosed with a health condition [35], and diagnosis and incidence of migraine and stroke does often depend on age [36, 37].

### Study design, data source, and sample

**Fig. 2:**
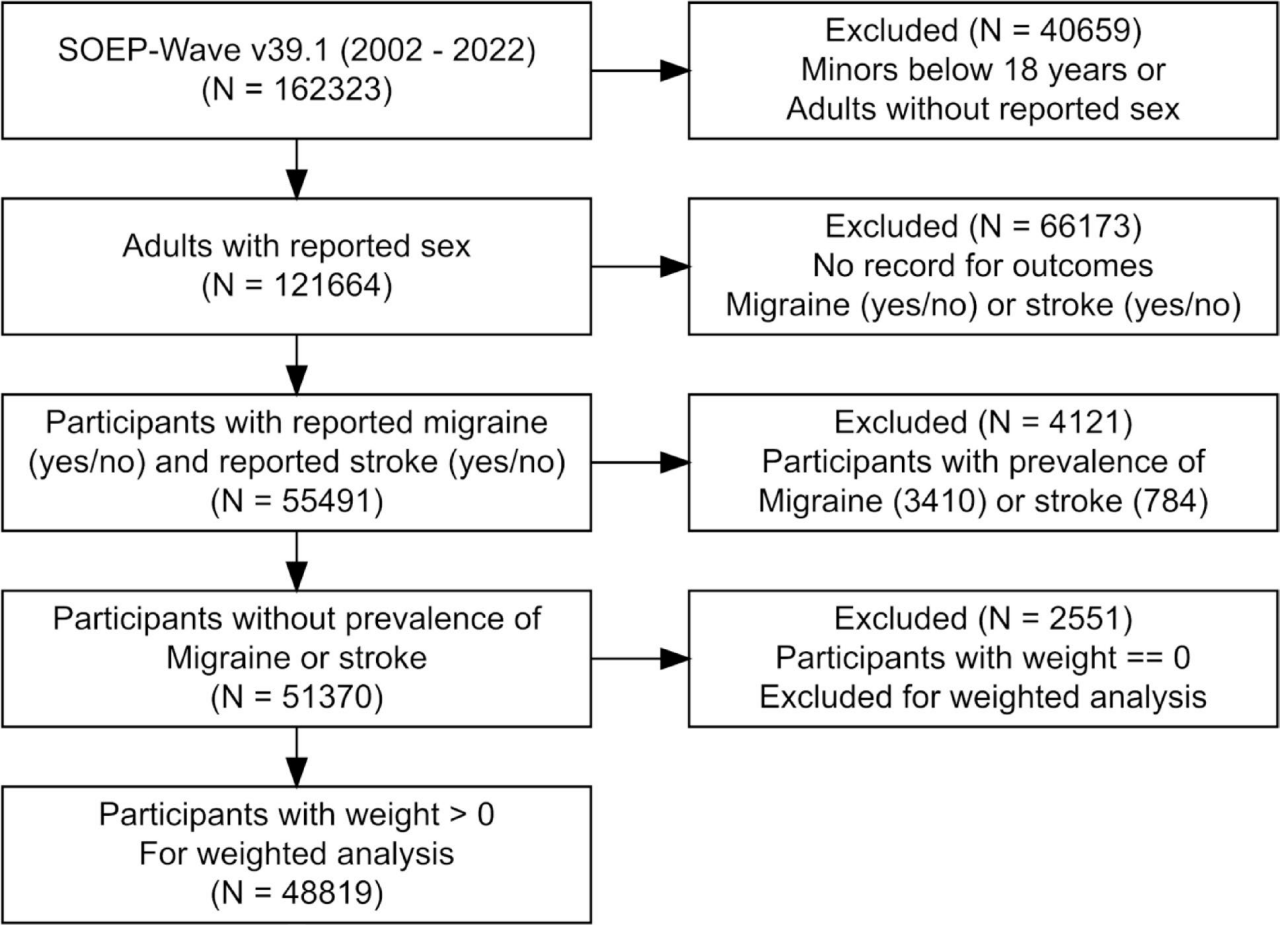
Flowchart of sample selection from the Socio-Economic Panel: SOEP-Core v39eu

The analysis is based on population data from 14 survey waves of the German Socio-Economic Panel (SOEP) between 2009 and 2022 [38]. As an annual panel that started in 1984 and is nationally representative, the SOEP polls more than 30,000 individuals in over 15,000 households and includes various socio-economic and health-related variables [23]. Information on self-reported diagnoses of a variety of health conditions is assessed biannually since 2009 with the question: ‘Has a doctor ever diagnosed you with one or more of the following diseases?’ with a range of conditions as possible answers. Individuals with valid answers to this question referring to migraine and stroke (either yes or does not apply for any of the diseases, implying no for migraine and for stroke) were selected into the study sample (see Flowchart in Fig. 2). For an analysis of main risk factors occurring before the outcome, we selected participants with recorded smoking (‘ever smoking’ or ‘current smoking’) from the first appearance in the dataset onwards from 2002. We included only individuals newly diagnosed after 2009 to calculate the incidence of newly diagnosed cases, excluding individuals with a (prevalent) diagnosis of migraine or stroke as their first valid response to these questions. Another inclusion criterion was age of at least 18 years. The resulting sample size was 51,370, of whom 48,819 had a non-zero representativeness weight. We applied a weighted analysis with wave-specific cross-sectional weights to account for varying respondent participation probabilities.

### Metrics

Detailed information on the assessment and recoding of all variables for the main and sensitivity analyses can be found in Additional file 1 Table S1. Age was computed based on ‘survey year’ and ‘birth year’ and included in the analysis as ‘age’ in years and ‘age group’ (18–34; 35–49; 50–64; 65-inf.). Sex assigned at birth was assessed as a binary variable including female and male. Gender-related variables serving as indicators for gender were selected based on prior research. Specifically, these were: ‘daily hours for housework on weekdays’ (continuous) [20, 39, 40], ‘daily hours for childcare on weekdays’ (continuous) [41], ‘current maternity or parental leave’ (dichotomous) [42], ‘gross hourly wage’ and ‘gross monthly labor income’ (in Euros) [40, 43], ‘highest educational degree’ (continuous, 0–8) [11], ‘employment status’ (continuous, 0–3) [29, 44], ‘risk-taking scale’ (continuous, 0–10) [41], ‘political interest’ (categorical, not at all/not so strongly/strongly/very strongly) [11]. We calculated the categorical variable ‘number of children in household’ (none/single/multiple) from information on whether underaged children lived in the same household as sample adult participants during any survey wave. The variable ‘partner’ indicated whether a person was living with a partner in the same household. ‘Sexual orientation’ was assessed in 2016 with a direct question. This information was complemented by biographic information available for all other years and individuals who have not answered the question and summarized into the categories ‘probably heterosexual’ and ‘probably bi- or homosexual’.

‘Current smoking’ and ‘ever smoking’ were assessed as binary variables (yes/no). For all participants, we systematically reviewed all years of data and documented when smoking, hypertension, diabetes, migraine, and stroke were first reported. We then identified when the outcome variables (diagnosis of migraine or stroke) first switched from 0 (no) to 1 (yes) for each participant. From this information, we created the variables ‘hypertension before stroke/migraine’, ‘diabetes before stroke/migraine’, and ‘smoking before stroke/migraine’.

The two outcome measures, ‘incidence of migraine diagnosis’ and ‘incidence of stroke diagnosis’, were specified as binary variables, whether a diagnosis was given (yes/no) between the last and the recent survey waves. As self-reported diagnoses of health conditions are only assessed biannually, only 6 study periods can be considered for the incidence rate (2011, 2013, 2015, 2017, 2019, 2021). The information from these waves was copied to the subsequent wave and not treated as missing in the analysis.

Finally, we included the variable ‘immigration history’ (categorical: ‘no’ when the individual and their parents were born in Germany; ‘direct’ immigration history when the individual was born outside of Germany and migrated themselves; ‘indirect’ immigration history when at least one parent of the individual was born outside of Germany). To create this variable, the SOEP combines information from the participants’ country of birth and (grand)parental information on their country of birth and their citizenship.

### Structural equation model (SEM)

We directly converted the DAG shown in Fig. 1 into the specification of a SEM model (Additional file 2) to test the model assumptions in the data, noting that gender needs to be modeled as a latent variable. Since our research question aimed at the direct effects of sex and gender, we did not include intermediate paths in the SEM to keep the model as parsimonious as possible.

SEM is a multivariate methodology introduced in the 1970s. It estimates causal links between variables based on a path diagram that includes both direct and indirect effects and combines the statistical methods *confirmatory factor analysis* and *path analysis*. The SEM is designed to study latent structures of complex (social) phenomena, indirectly measuring latent variables while considering the relationships with observable variables [45, 46].

### Statistical analysis

To reduce the number of records with missing values, variables that were generated by the SOEP panel using all information available for most participants from all possible time points were selected if suitable (‘employment status’, ‘gross monthly income’, ‘immigration history’). Missing information on the gender-related variables or potential risk factors for the outcomes for inclusion in the main models varied between less than 0.1% and 24.5%. We investigated the patterns of missingness to assess whether data were missing at random (MAR) (Additional file 3). Following the approach of Grochtdreis, König, and Dams [47], we used multiple imputation by chained equations (MICE) with predictive mean matching for continuous data, logistic regression-based imputation for dichotomous data, and proportional odds logistic regression for ordinal categorical variables, with a total number of m = 20 imputations. The health outcomes, gender-related, sociodemographic and migration-related variables were included in the imputation model.

We compiled descriptive statistics of population characteristics and all relevant variables, showing mean values and standard deviations (SD) for continuous data and frequencies and proportions for categorical data.

For the main analysis, we applied SEM to simultaneously calculate gender as a latent variable and test the hypothesized relationships from the DAG using maximum likelihood estimation. We specified the measurement model to calculate the latent variable ‘gender’ based on the preselected gender-related variables. We also modeled gender to be influenced by ‘sex binary’. As we hypothesized that the relationship between sex and gender was modified by ‘age’ [29] and ‘immigration history’ [48], we included an additional analysis stratified by ‘age group’. We scaled the latent gender variable so that its arbitrary scale matches that of ‘sex’ to be able to directly compare the effect estimates of sex and gender, thereby forcing the mean gender for females to 1 and for males to 0. We plotted the resulting gender distributions stratified by ‘sex’ and ‘age group’ (see Additional file 4), and by ‘sex’ and ‘immigration history’ (see Additional file 5). To ensure that the choice of scaling gender to match sex on average was not overly prescriptive, we added a sensitivity analysis using the same SEM but removing the link between gender and sex altogether, so that gender was estimated solely from the gender-related variables (see Additional files 2 and 11).

The models included the respective outcome as dependent variable, ‘newly diagnosed migraine/stroke’, and the following independent variables: ‘sex binary’, ‘gender’, ‘sexual orientation’, ‘partner’, ‘age 10years’, ‘smoke before migraine/stroke’, ‘diabetes before migraine/stroke’, and ‘hypertension before migraine/stroke’. Statistical significance of coefficients with confidence intervals (CI) was assessed based on t-values (|t| > 1.96) and corresponding p-values (p < .05). The main analysis was conducted based on the unweighted sample.

We performed additional analyses using the ‘age group’ stratified (see Additional file 6 Table S2) or the weighted samples (see Additional file 7 Table S3). Furthermore, we explored alternative models using a causal discovery technique [49], including all variables except for the weight factors, separately for all imputed datasets and for the raw data only (see Additional file 8).

All analyses were conducted using R 4.5.2 [50], RStudio 2026.01.0 [51], mice 3.19.0 [52], and lavaan 0.6–21 packages [53]. The R script files are publicly available via GitHub.

## Results

### Sociodemographic and gender-related characteristics

The total sample comprised 51,370 individuals (49.9% of whom reported they were assigned female at birth), cf. Table 1. The mean age was 50 years and the majority were living in a partnership (55.1%). Most participants had no children living in the same household (69.0%) and the median highest educational degree was upper secondary education. The majority were German born with no immigration history (72.5%).

Regarding the gender-related variables (see Additional file 9 Table S5), female participants on average spent more time on both housework (Mean = 2.16 vs. 0.98 h) and childcare on weekdays (Mean = 1.85 vs. 0.64 h), were more likely to be unemployed (42.7% vs. 33.8%) or working part-time (29.4% vs. 8.6%), and had an average monthly gross labor income that was €1,610 less compared to male persons. Female individuals were also more likely to be on parental leave (3.9% vs. 0.4%), reported lower risk-taking, lower levels of political interest, and slightly more frequent physician visits.

Female persons were less likely to have smoked before stroke (34.0% vs. 45.4%) or migraine onset (33.9% vs. 45.3%) and reported slightly lower pre-existing diabetes (7.6% vs. 8.9% before stroke and 7.4% vs. 9.0% before migraine) and hypertension diagnoses (25.9% vs. 28.4% before stroke and 25.2% vs. 28.3% before migraine).

**Table 1:** Population Characteristics

	Male sex (*N* = 25741)	Female sex (*N* = 25629)	Overall (*N* = 51370)
**Age**			
Mean (SD)	50.1 (18.4)	50.3 (18.5)	50.2 (18.5)
Median [Min, Max]	50.0 [18.0, 106]	50.0 [18.0, 104]	50.0 [18.0, 106]
**Age Group**			
18–34	6359 (24.7%)	5997 (23.4%)	12,356 (24.1%)
35–49	6242 (24.2%)	6796 (26.5%)	13,038 (25.4%)
50–64	7066 (27.5%)	6697 (26.1%)	13,763 (26.8%)
65-Inf	6074 (23.6%)	6139 (24.0%)	12,213 (23.8%)
**Sexual orientation**			
Probably Heterosexual	21,196 (82.3%)	21,186 (82.7%)	42,382 (82.5%)
Probably Bi- or Homosexual	533 (2.1%)	590 (2.3%)	1123 (2.2%)
Missing	4012 (15.6%)	3853 (15.0%)	7865 (15.3%)
**Living with partner in household**			
No	10,396 (40.4%)	11,139 (43.5%)	21,535 (41.9%)
Yes	14,591 (56.7%)	13,738 (53.6%)	28,329 (55.1%)
Missing	754 (2.9%)	752 (2.9%)	1506 (2.9%)
**Number of children in household**			
None	18,128 (70.4%)	17,304 (67.5%)	35,432 (69.0%)
Single	3397 (13.2%)	3877 (15.1%)	7274 (14.2%)
Multiple	4216 (16.4%)	4448 (17.4%)	8664 (16.9%)
**Immigration history**			
None	18,572 (72.1%)	18,650 (72.8%)	37,222 (72.5%)
Indirect	1632 (6.3%)	1599 (6.2%)	3231 (6.3%)
Direct	5537 (21.5%)	5380 (21.0%)	10,917 (21.3%)
**Migraine incidence**			
No	25,218 (98.0%)	24,036 (93.8%)	49,254 (95.9%)
Yes	523 (2.0%)	1593 (6.2%)	2116 (4.1%)
**Stroke incidence**			
No	25,303 (98.3%)	25,325 (98.8%)	50,628 (98.6%)
Yes	438 (1.7%)	304 (1.2%)	742 (1.4%)

### Descriptive statistics of health outcomes

Migraine incidence was 3.1 times higher among female participants (6.2% vs. 2.0%), while stroke incidence was lower (1.2% vs. 1.7%). The cumulative incidence of participants who were newly diagnosed with migraine between 2011 and 2022 was 4.1% in the total sample (2,116 individuals, 1,593 female, 523 male, reported being newly diagnosed). The cumulative incidence for stroke between 2011 and 2022 was 1.4% (742 individuals, 304 female, 438 male, reported a new diagnosis during this time period).

### Structural equation model

**Fig. 3:**
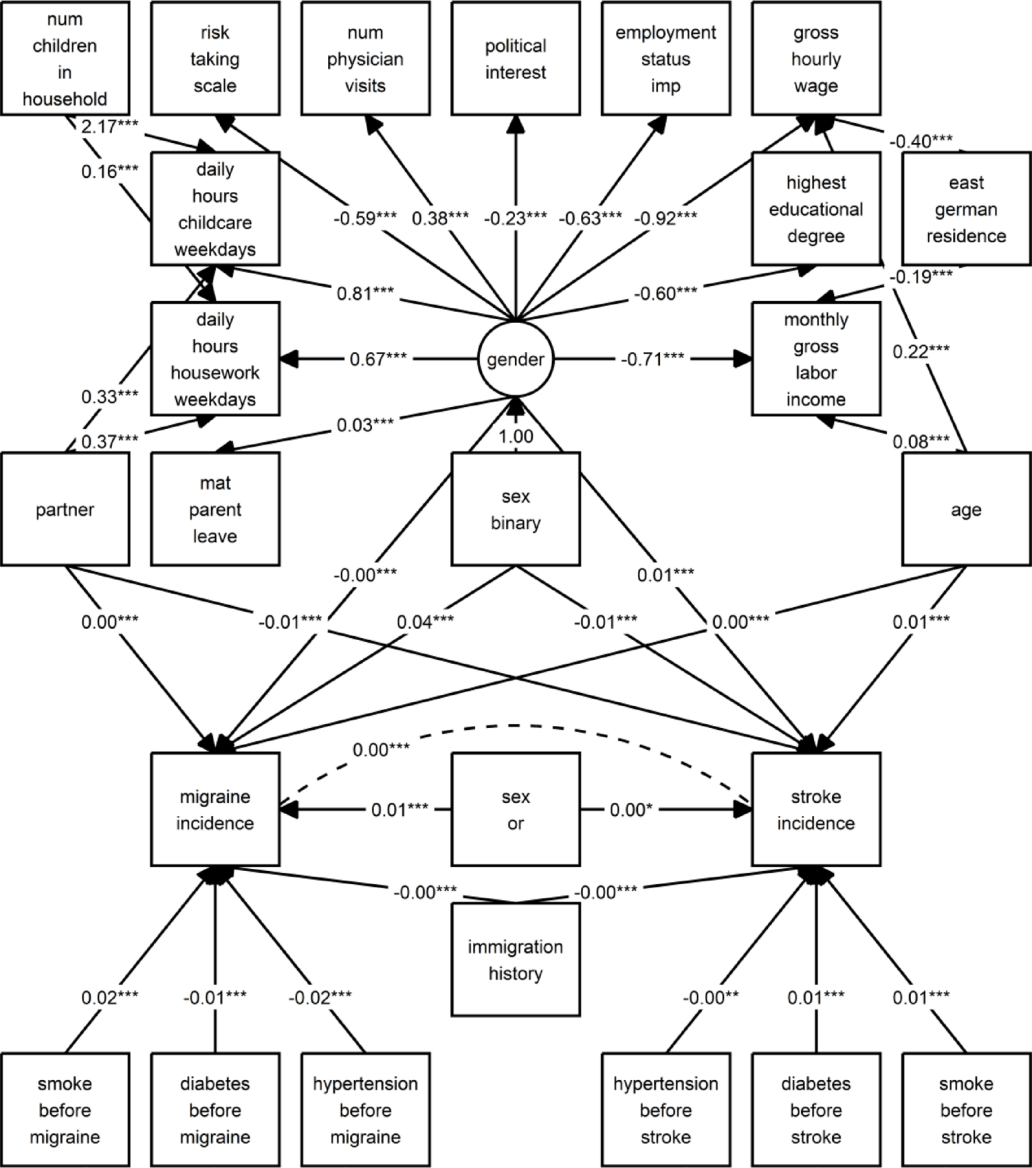
Structural equation model (SEM) plot displaying standardized path coefficients for the hypothesized relationships outlined in Fig. 1 (from the main SEM for all imputation instances combined)

All observed indicators in the measurement model were statistically significantly reflecting the latent variable gender (see Fig. 3). The variable ‘daily hours of housework’ was the strongest gender indicator (*β* = 0.670, 95% CI [0.619, 0.722], *t* = 26.458, *p* < .001) with a positive probit, i.e., higher values on the gender spectrum for every one hour increase in housework per day. The other gender-related variables with positive coefficients were ‘daily hours of childcare’, ‘current maternity or parental leave’, and ‘number of physician visits in the last three months’. The gender-related variables with negative coefficients, implying lower values on the gender spectrum with a one-unit increase, are ‘current gross monthly income’, ‘gross hourly wage’, ‘employment status’, ‘highest educational degree’, ‘risk-taking scale’, and ‘political interest’. The strongest gender indicator with a negative probit was ‘current monthly income’, with (*β* = -0.708, 95% CI [-0.756, -0.661], *t* = -29.130, *p* < .001). The entire SEM output containing the estimates, confidence intervals, t-values, and p-values can be seen in Additional file 10.

The structural component of the model in Fig. 3 estimated the direct effects of sex and gender, as well as the associations between potential risk factors and new migraine and stroke diagnoses. The strongest effect on migraine incidence was shown for ‘binary sex’. In the main analysis, respondents of female sex were more likely to receive a new migraine diagnosis during the study period than respondents of male sex (*β* = 0.044, 95% CI [0.033, 0.056], *t* = 7.734, *p* < .001). Additional file 7 Table S3 shows the results of the structural model for the unweighted main and the weighted analyses, as well as for the sensitivity analysis. Overall, the results were very consistent between all three models.

In all models, the coefficients for the effect of gender on migraine incidence showed no direct effect. A probably bi- or homosexual orientation compared to probably heterosexual showed an insignificant positive association with migraine incidence, while living with a partner compared to being single was not associated. A 10-year increase in age showed a small and statistically significant increase in migraine diagnosis with age, and smoking was strongly and significantly associated with the risk for a subsequent migraine diagnosis. The regression coefficients for diabetes and hypertension before migraine diagnosis were both negative, suggesting a prior diagnosis of diabetes or hypertension or their medical management to be protective for a migraine diagnosis (with the effect of ‘diabetes before migraine’ only being statistically significant in the weighted analysis).

In all models for stroke incidence, sex showed a negative statistically significant direct effect on stroke, indicating that slightly fewer female persons reported a new stroke diagnosis (*β* = -0.010, 95% CI [-0.017, -0.004], *p* < .01; *β*= -0.013, 95% CI [-0.018, -0.007], *p* < .001). The coefficient for gender suggested a small and significant increase in reporting a stroke diagnosis with higher gender values (*β* = 0.006, 95% CI [0.002, 0.009], *p* < .01, *β* = 0.006, 95% CI [0.004, 0.009], *p* < .001). Living with a partner was negatively significantly associated with reporting of stroke diagnosis (*β* = -0.008, 95% CI [-0.013, -0.002], *p* < .05; *β* = -0.008, 95% CI [-0.013, -0.002], *p* < .01). These three relations were also statistically significant in the sensitivity analysis, with the same effect directions and identical or similar effect sizes. Older age and pre-stroke smoking showed strong statistically significant associations with increased stroke incidence, while the strong association for diabetes was not statistically significant. In contrast, sexual orientation, immigration history, and hypertension were not statistically significantly associated with stroke diagnosis in either of the models.

The results for the age group stratified analyses can be found in Additional file 6. For migraine incidence, all coefficients for the separate age groups are positive for female sex. They steadily increase with age showing the largest effect on migraine incidence reporting in the oldest age group from 65 years on. The results for gender are less consistent with slight negative tendencies for older age groups, a null value for the youngest group, and a positive tendency only in the age group of 35–49 years, suggesting a small effect of positive gender values linked to characteristics mainly reported by female persons on migraine incidence. Sexual orientation shows a small positive association in the youngest group of 18–34 years and the two oldest groups, but not the one of 35-49-year-olds. Results for smoking were consistently positive for all age groups; all coefficients for diabetes prior to migraine diagnosis were negative. Hypertension before migraine was absent in the youngest age group and had negative associations with migraine incidence for all other age groups.

For stroke incidence, the results showed that male sex had the strongest effect in the oldest age group and was null for the youngest. Positive gender values had positive effects on stroke diagnosis in all age groups except for the youngest and showed the largest coefficient for the oldest group. Results for sexual orientation were inconsistent, and living in partnership was not associated in the youngest age group with stroke incidence but was negatively associated with reporting of stroke in all other groups with the largest effect for the oldest age group. Smoking showed a positive association with stroke for all groups, as did diabetes, except for the youngest group. Coefficients for hypertension were small and positive, and only small and negative for the oldest age group.

The Root Mean Square Error of Approximation, Standardized Root Mean Square Residual, and Akaike Information Criterion were used as fit indices for all models, which can be seen in Additional file 7 Table S4. They suggested a good fit for the main model and the best fit for the age stratified model.

We plotted the latent gender value by ‘sex’ and ‘age group’ or by ‘sex’ and ‘immigration status’ to assess socialization differences (Additional files 4 and 5). In the youngest age groups for the whole sample and the group born in Germany or immigrated as children below 6 years old, the gender distributions plotted for male and female individuals largely overlapped, indicating minimal sex differences in gender-related characteristics.

The causal discovery (see Additional file 8) using all imputed datasets revealed an additional relationship between ‘using a period of care’ and gender, while the discovery using only the raw data found a link between ‘work time’ and gender, which had both been identified as potential gender related variables, but not included in the SEM due to collinearity with other included variables.

## Discussion

In this longitudinal study, we applied a causal framework and SEM to estimate gender as a latent variable and study the effects of sex and gender on migraine and stroke diagnoses within the same model using population-representative German panel data. Our results demonstrated that both sex assigned at birth and gender — operationalized as a continuous latent construct shaped by sex and multiple socio-economic and behavioral indicators — independently and differentially contributed to stroke incidence between 2011 and 2022. The picture is more complex for migraine, where only sex showed a statistically significant effect, while no direct effect of gender was found in the main analysis. However, differences in gender effects were seen in the stratified analysis of the age groups.

Specifically, being assigned female at birth showed the strongest effect on migraine incidence compared to other contributing factors. Migraine incidence was 3.1 times higher in female than in male participants, which is consistent with established evidence [1]. However, gender was not found to statistically significantly affect being newly diagnosed with migraine in the main analysis. Previous studies have described a relationship between risk of migraine and high levels of daily stress, suggesting a possible effect of gender aspects [1, 54]. Since stress can be viewed as a mediator for the effect of gender (defined by several of the gender-related variables we chose: more time use for childcare and household chores and lower socio-economic status, which are characteristics usually linked to women), we expected to see that higher gender values would increase migraine incidence. This association was only found in the early middle-aged group, which may be due to the increased psychosocial stress due to caregiving burdens [55], which are more relevant for individuals in midlife. Ballering et al. found that both female sex and feminine gender were associated with higher migraine lifetime prevalence using a gender score, with sex being almost twice as strongly associated with migraine than gender [20].

Besides sex and gender effects, we looked at several other sex- and/or gender-related factors that may be associated with migraine and/or stroke: Although not statistically significant in our sample, non-heterosexual orientation was associated with a higher incidence of migraine diagnosis, confirming results based on the SOEP data combined with a boost sample of lesbian, gay, bisexual, trans, queer, and inter persons [56], possibly suggesting an impact of minority stress on migraine [57]. Having a partner was not associated with migraine diagnosis, but protective of reporting a new stroke diagnosis, especially in the older age group, where individuals are more likely to live alone or be widowed, especially women who have a higher life expectancy [3]. This points to the gendered component in the importance of the social and daily living context in development and diagnosis of stroke [3].

In our study, individuals who smoked before their migraine or stroke diagnoses were more likely to be diagnosed with both conditions, especially migraine. In general, the causal link between smoking and migraine is unclear [58, 59]. People with migraines may be more likely to smoke [60], pointing to possible reverse causation rather than the association we have investigated. Smoking has been discussed as one of the top stroke-related risk factors in high income countries, based on data from the Global Burden of Disease Study [61]. Hypertension and diabetes have been reported as important pre-stroke risk factors, especially in the context of socioeconomic disparities [62] and sex differences [3]. Both were not associated with stroke incidence in our model that included socioeconomic factors in the latent gender variable, besides sex. Possibly age, sex, and gender explained most of the effect in our sample. However, previous studies suggest that medication against hypertension, which we had no data on, may influence stroke incidence [63]. Hypertension before migraine in our study suggested a protective association with migraine incidence diagnosis. Blood pressure-lowering medication was reported to reduce migraine/headache frequency in migraine patients [64], possibly explaining our finding. However, the causal relationship between hypertension and migraine may be reverse, with existing evidence that hypertension is a consequence of migraine rather than a cause [65].

Regarding the effect of sex and gender on stroke, participants assigned male at birth were more likely to report new diagnoses, confirming existing knowledge that stroke incidence rates are higher for men compared to women in Germany [66]. Prior studies on sex differences in stroke have rarely distinguished sex from gender, thus reported sex differences are most likely due to both sex and gender aspects [3]. For example, a strong educational gradient was found for adult women with a threefold higher stroke prevalence in the lower education group [67], suggesting the impact of gendered socio-economic factors. Our study found that higher levels on the latent gender spectrum, associated with lower education and income, but higher hours of housework and childcare, among others, had an effect on reporting more stroke cases. Thus, sex and gender had differential effects on stroke incidence, although the gender effect was weaker. This finding supports our prior hypothesis. It is consistent with results of Ballering et al., using the gender score, showing that female sex was associated with lower and feminine gender with higher stroke prevalence [20].

Similar to our approach, the gender score method estimates a latent gender variable [15] resulting in a unidimensional, bipolar continuum between “femininity” and “masculinity”, which has been critically discussed elsewhere [2, 13, 41]. Yet, differently to the gender score method, our approach allows a different interpretation of the continuous gender variable: Our age-group stratified analysis not only showed that gender effects may vary within different age groups, but also shows variation within distribution of the gender variable itself. Plotting the gender variable by sex and age group revealed that especially for young female individuals aged between 18 and 34, higher educational degree and gross hourly wage was not statistically significantly associated with lower gender values more often shown by male individuals, like in older age groups. While in data-driven gender scores, only those gender-related variables that predict female or male sex can be chosen, the SEM approach allows more flexibility in the inclusion of gender variables and the interpretation of the gender scale. Higher or lower gender values do not necessarily have to be interpreted as “femininity” or “masculinity” and their distribution can vary among different groups. The theory-driven variable selection can be adapted to relevant gender dimensions and to specific contexts, to account for the time- and context-specificity of gender [7].

Another theory-driven approach based on gender-related variables, which refrains from coding variables as “masculine” or “feminine”, is the Stanford Gender-Related Variables for Health Research tool [41]. Similar to the gender score approach, associations of the gender-related variables with health outcomes in this method can only be studied in singular regression models. Furthermore, as sex assigned at birth and gender identity were too highly correlated in the Stanford approach, all models were run twice with each variable alternately included as main predictor [41]. Modelling latent gender as a consequence of sex, our SEM approach allows studying the effects of both variables on health outcomes together in the same model. To the best of our knowledge, only some of the strategies explored by Colineaux et al. [21] include both sex and gender in the same model using g-computation to study health effects.

Nevertheless, it cannot be concluded that the effects of sex and gender on health outcomes can be strictly separated [2]. This is also true in our SEM approach. We defined gender to be a descendant of sex, acknowledging the influence of sex on gender-related variables and thereby the latent gender construct. Furthermore, sex may affect migraine and stroke as well as being diagnosed with both via other socio-cultural and behavioral factors which we have not included in our analysis, for instance physical activity, diet, medication, chronic stressors like discrimination [1, 21], or gendered utilization or access to care [68]. Our estimated effect of sex thus may still include the effect of gendered pathways.

Recent discussions have highlighted that sex and gender are interrelated, and that whether their effects on health can be disentangled depends on the conceptualization of the sex-gender-relationship [13]. Previous research has established that causal knowledge and causal diagrams can be useful to conceptualize the relationship between sex and gender [2, 15]. We combined a causal framework with an SEM to show that sex and gender differentially impact migraine and stroke incidence.

### Strengths and limitations

Using a DAG, we demonstrated that sex and gender are two distinct, but interrelated concepts that may have different effects on health, depending on their definitions. Our approach allows modeling the complexity of social reality, where biological and social aspects of sex and gender can be theoretically conceptualized together in a DAG and modeled within the same model using SEM. This approach is useful to help understand the mechanisms that may contribute to sex differences in health outcomes. To the best of our knowledge, this study is the first to conceptualize and measure gender as a latent variable in an SEM, which is a flexible and versatile technique that can be adapted to different socio-cultural contexts and different gender definitions or available variables. Another clear advantage of SEM over gender scores, which are based on logistic regression, is the possibility to include a non-dichotomous exogenous sex variable.

However, our results should be interpreted with caution. The SEM is based on key assumptions such as normality of the noise terms, no multicollinearity, and linear relationships between the variables [46]. Some of these assumptions could not be verified or are not met in our model, including the normal distribution of the latent gender variable. The effect of sex in our example cannot be interpreted as merely biological. We do not have any information about which sex-related aspects drive the effect – e.g., hormonal or genetic factors, having only data on sex assigned at birth. Part of the sex differences could still be explained by socio-behavioral mechanisms [21] not included in the model.

Our study was limited by the availability, completeness, and type of variables in the SOEP panel. Sex assigned at birth was assessed as dichotomous, not taking intersex variations and recent official categories in Germany (diverse and no entry) into account. Self-identification of gender identity was not included in the dataset. All health conditions were assessed as self-reported diagnoses, possibly introducing bias by not accounting for differences in access or utilization of healthcare services. Several variables were not available or were not included that would have been relevant for our research question, such as hormone levels, use of oral contraceptive pills, medication, alcohol consumption, dyslipidemia, obesity, physical activity, and stress.

Due to the scope of our paper, we were unable to take socialization differences regarding immigration history or early-life social class into account in a more nuanced analysis. Intersectional analyses should be further explored, including a variety of diversity categories that may shape gendered socialization processes.

With sex and gender as exposures, we aimed to measure the causal effect of non-intervenable variables, contributing to a discussion that has been ongoing [69, 70]. Future perspectives could focus on mediation analyses within SEM models using a causal framework which may open up possibilities for intervenable pathways [71, 72]. Including mediation analyses to the SEM latent gender model would furthermore allow for more nuanced analyses of additional relevant behavioral factors as well as for estimating the total effect of gender on health outcomes.

## Conclusion

Integrating a causal framework with an SEM, our study proposes an alternative method to investigate concurrent sex and gender health effects, acknowledging the interplay of biological and sociocultural factors. Sex assigned at birth and the latent gender variable both contributed to stroke incidence in the German panel between 2011 and 2022, through different and common pathways, whereas only sex showed a direct effect on migraine. Further exploring sex and gender effects in health research beyond binary categorization is critical for informing more equitable, inclusive, and effective healthcare interventions.

## Supplementary Information

Below is the link to the electronic supplementary material.

Supplementary Material 1

Supplementary Material 2

Supplementary Material 3

Supplementary Material 4

Supplementary Material 5

Supplementary Material 6

Supplementary Material 7

Supplementary Material 8

Supplementary Material 9

Supplementary Material 10

Supplementary Material 11

## Data Availability

The data can be accessed by submitting a user-contract application to the SOEP Research Data Center: [https://www.diw.de/en/diw_01.c.601584.en/data_access.html](https:/www.diw.de/en/diw_01.c.601584.en/data_access.html)The R script files are publicly available via GitHub: [https://github.com/wischmha/LatentGenderSEM](https:/github.com/wischmha/LatentGenderSEM).

## Declarations

The purpose of the data use to which the participants have consented includes the present analysis: https://www.diw.de/en/diw_01.c.601584.en/data_access.html#c_diw_01.c.741347.de.
