# Supplementary material for: Sex and gender effects on incidence of migraine and stroke: a longitudinal observational study based on the german socio-economic panel
Source: Biol Sex Differ. 2026 Mar 16;17:73. doi: 10.1186/s13293-026-00875-z (PMC13064216; doi:10.1186/s13293-026-00875-z)
Supplement: Supplementary file 1 — Supplementary Material 1 [file 13293_2026_875_MOESM1_ESM.docx]

## Table S1. Recoding of all variables for the analyses

| Variable | Name | Values | Mappings |
| --- | --- | --- | --- |
| hid | hid |  |  |
| sex_binary | sex | 1 (male) 2 (female) -1 (no answer) -2 (does not apply) -3 (answer improbable) | 1 > 0 2 > 1 <0 > NA |
| birth_year | gebjahr | 1909 … 1972 … 1984 … | <0 > NA |
| immi_year | immiyear | 1950 … | <0 > NA |
| immigration_history | migback | 1 (no mig back) 2 (direct mig back) 3 (indirect mig back) -1 (no answer) | 1 > 0 3 > 1 <0 > NA |
| refugee_exp | arefback | 1 (no evidence) 2 (with evidence of direct ref exp) 3 (with evidence of indirect ref exp) -1 (no answer) | 1 > 0 3 > 1 <0 > NA |
| east_german_residence | sampreg | 1 (West-G) 2 (East-G) -1 (no answer) -2 (does not apply) | 1 > 0 2 > 1 <0 > NA |
| sex_or | sexor | 0 (probably hetero) 1 (probably bi or homo) 2 (insufficient info) | 2 > NA |
| partner | partner | 0 (no partner) 1 (spouse or registered partner) 2 (partner) 3 (probably spouse or registered pertner) 4 (probably partner) 5 (not clear) -1 (no answer) -2 (does not apply) | 2 > 1 3 > 1 4 > 1 5 > NA <0 > NA |
| sex_entry_change | pla0048 | 1 (yes) 2 (no) -1 (no answer) -5 (not included) -8 (question this year not part of survey) | 1 > 0 2 > 1 <0 > NA |
| felt_discriminated_gender_12mo | plh0415i03 | 1 (yes) 2 (no) | 2 > 0 <0 > NA |
| felt_discriminated_ethnic_12mo | plh0415i01 | 1 (yes) 2 (no) | 2 > 0 <0 > NA |
| using_period_of_care | plb0020_h | 1 (yes) 2 (no) | 2 > 0 <0 > NA |
| current_mat_parent_leave | plb0019_v2 | 1 (yes maternity) 2 (yes parental) 3 (no) | 3 > 0 2 > 1 <0 > NA |
| risk_taking_scale | plh0204_v2 | 0-10 | <0 > NA |
| risk_taking_driving_scale | plh0197 | 0-10 | <0 > NA |
| risk_taking_health_scale | plh0201 | 0-10 | <0 > NA |
| highest_educational_degree | pgisced11 | 0 (in school) 1 (primary education) 2 (lower secondary eduation) 3 (upper secondary education) 4 (post-secondary non-tertiary education) 5 (short-cycle tertiary) 6 (Bachelors or equivalent) 7 (Masters or equivalent) 8 (Doctoral or equivalent) | <0 > NA |
| current_monthly_gross_labor_income | pglabgro | Euros gross | <0 > NA |
| daily_hours_childcare_weekdays | pli0044_h | 0-80 | <0 > NA |
| daily_hours_housework_weekdays | pli0043_h | 0-84 | <0 > NA |
| employment_status_imp | pgemplst | 1 (full-time) 2 (regular part-time) 3 (vocational training) 4 (irregular part-time) 5 (not employed) 6 (sheltered workshop) 7 (short-time work) | 5 > 0 1 > 13 3 > 1 6 > 1 4 > 2 7 > 2 13 > 3 <0 > NA |
| work_time | pgtatzeit | 0-80 | <0 > NA |
| leadership_position | plb0067 | 1 (yes) 2 (no) -1 (no answer) -5 (not included) -8 (question this year not part of survey) | 2 > 0 <0 > NA |
| frequency_homeoffice | plb0096_v1 | 1 (daily) 2 (several times per week) 3 (once every 2 to 4 weeks) 4 (rarely) -1 (no answer) -2 (does not apply) -5 (not included) -8 (not part of syear) | -2 > 0 1 > 2 3 > 1 4 > 1 <0 > NA |
| working_overtime | plb0193_v1 | 1 (yes) 2 (no) 3 (does not apply self-employed) -1 (no answer) -2 (does not apply) | -2 > 0 2 > 0 3 > 2 <0 > NA |
| worried_health | plh0035 | 1 (big worries) 2 (some worries) 3 (no worries) -1 (no answer) -2 ( does not apply) | <0 > NA |
| worried_pension | plh0335 | 1 (big worries) 2 (some worries) 3 (no worries) -1 (no answer) -2 ( does not apply) | <0 > NA |
| career_sacrifices | plb0115 | 1 (Strongly disagree) 2 (Rather disagree) 3 (Rather agree) 4 (Fully agree) -1 (No answer) -2 (Does not apply) | <0 > NA |
| current_life_satisfaction_scale | plh0182 | 0-10 | <0 > NA |
| political_interest | plh0007 | 1 (Very strongly) 2 (strongly) 3 (not so strongly) 4 (not at all) -1 (no answer) -2 (does not apply) | 4 > 0 1 > 4 3 > 1 4 > 3 <0 > NA |
| current_health | ple0008 | 1 (very good) 2 (good) 3 (satisfactory) 4 (less good) 5 (bad) -1 (no answer) | <0 > NA |
| self_esteem | plh0206i11 | 1-7 | <0 > NA |
| health_insurance_status | ple0099_v5 | 1 (Pflichtmitglied) 2 (freiwillig) 3 (mitversichters Familienmitglied) 4 (versichert als Rentner) -1 (no answer) -2 (does not apply) -3 (implausible) -4 (multiple response) -5 (not included) -8 (not in syear) | <0 > NA |
| alcohol_consumption | ple0177 | 1 (daily) 2 (4 to 6 days) 3 (2 to 3 days) 4 (2 to 4 days) 5 (once a month or less) 6 (never) | 6 > 0 5 > 0 1 > 2 4 > 1 3 > 1 <0 > NA |
| current_smoke | ple0081_v2 | 1 (yes) 2 (no) -1 (no answer) | 2 > 0 <0 > NA |
| ever_smoke | ple0080_v3 | 1 (yes) 2 (no) -1 (no answer) | 2 > 0 <0 > NA |
| num_physician_visits | ple0072 | 0-99 | <0 > NA |
| diabetes | ple0012 | 1 (Yes) -1 (no answer) -2 (does not apply) | -2 > 0 -1 > 0 <0 > NA |
| hypertension | ple0018 | 1 (Yes) -1 (no answer) -2 (does not apply) | -2 > 0 -1 > 0 <0 > NA |
| migraine | ple0017 | 1 (Yes) -1 (no answer) -2 (does not apply) | -2 > 0 -1 > 0 <0 > NA |
| stroke | ple0016 | 1 (Yes) -1 (no answer) -2 (does not apply) | -2 > 0 -1 > 0 <0 > NA |
| no_illness | ple0024 | 1 (Yes) -1 (no answer) -2 (does not apply) | -2 > 0 -1 > 0 <0 > NA |
| weight_factor | phrf |  |  |

## 
