# Supplementary material for: Sex and gender effects on incidence of migraine and stroke: a longitudinal observational study based on the german socio-economic panel
Source: Biol Sex Differ. 2026 Mar 16;17:73. doi: 10.1186/s13293-026-00875-z (PMC13064216; doi:10.1186/s13293-026-00875-z)
Supplement: Supplementary file 2 — Supplementary Material 2 [file 13293_2026_875_MOESM2_ESM.docx]

## Table S5. Population characteristics (current/most recent wave)

|  | **Sex_M (N=25741)** | **Sex_F (N=25629)** | **Overall (N=51370)** |
| --- | --- | --- | --- |
| **age** |  |  |  |
| Mean (SD) | 50.1 (18.4) | 50.3 (18.5) | 50.2 (18.5) |
| Median [Min, Max] | 50.0 [18.0, 106] | 50.0 [18.0, 104] | 50.0 [18.0, 106] |
| **age_group** |  |  |  |
| [18,35) | 6359 (24.7%) | 5997 (23.4%) | 12356 (24.1%) |
| [35,50) | 6242 (24.2%) | 6796 (26.5%) | 13038 (25.4%) |
| [50,65) | 7066 (27.5%) | 6697 (26.1%) | 13763 (26.8%) |
| [65,Inf) | 6074 (23.6%) | 6139 (24.0%) | 12213 (23.8%) |
| **sex_or** |  |  |  |
| Prob. Hetero | 21196 (82.3%) | 21186 (82.7%) | 42382 (82.5%) |
| Prob. Bi or Homo | 533 (2.1%) | 590 (2.3%) | 1123 (2.2%) |
| Missing | 4012 (15.6%) | 3853 (15.0%) | 7865 (15.3%) |
| **partner** |  |  |  |
| No | 10396 (40.4%) | 11139 (43.5%) | 21535 (41.9%) |
| Yes | 14591 (56.7%) | 13738 (53.6%) | 28329 (55.1%) |
| Missing | 754 (2.9%) | 752 (2.9%) | 1506 (2.9%) |
| **smoke** |  |  |  |
| No | 16063 (62.4%) | 18112 (70.7%) | 34175 (66.5%) |
| Yes | 7266 (28.2%) | 5399 (21.1%) | 12665 (24.7%) |
| Missing | 2412 (9.4%) | 2118 (8.3%) | 4530 (8.8%) |
| **hypertension** |  |  |  |
| No | 19631 (76.3%) | 20054 (78.2%) | 39685 (77.3%) |
| Yes | 6093 (23.7%) | 5557 (21.7%) | 11650 (22.7%) |
| Missing | 17 (0.1%) | 18 (0.1%) | 35 (0.1%) |
| **diabetes** |  |  |  |
| No | 23635 (91.8%) | 23938 (93.4%) | 47573 (92.6%) |
| Yes | 2089 (8.1%) | 1673 (6.5%) | 3762 (7.3%) |
| Missing | 17 (0.1%) | 18 (0.1%) | 35 (0.1%) |
| **migraine** |  |  |  |
| No | 25405 (98.7%) | 24615 (96.0%) | 50020 (97.4%) |
| Yes | 319 (1.2%) | 996 (3.9%) | 1315 (2.6%) |
| Missing | 17 (0.1%) | 18 (0.1%) | 35 (0.1%) |
| **stroke** |  |  |  |
| No | 25382 (98.6%) | 25373 (99.0%) | 50755 (98.8%) |
| Yes | 342 (1.3%) | 238 (0.9%) | 580 (1.1%) |
| Missing | 17 (0.1%) | 18 (0.1%) | 35 (0.1%) |
| **num_children_in_household** |  |  |  |
| None | 18128 (70.4%) | 17304 (67.5%) | 35432 (69.0%) |
| Single | 3397 (13.2%) | 3877 (15.1%) | 7274 (14.2%) |
| Multiple | 4216 (16.4%) | 4448 (17.4%) | 8664 (16.9%) |
| **daily_hours_childcare_weekdays** |  |  |  |
| Mean (SD) | 0.644 (1.75) | 1.85 (4.14) | 1.25 (3.23) |
| Median [Min, Max] | 0 [0, 24.0] | 0 [0, 24.0] | 0 [0, 24.0] |
| Missing | 28 (0.1%) | 23 (0.1%) | 51 (0.1%) |
| **daily_hours_housework_weekdays** |  |  |  |
| Mean (SD) | 0.979 (0.970) | 2.16 (1.54) | 1.57 (1.42) |
| Median [Min, Max] | 1.00 [0, 20.0] | 2.00 [0, 24.0] | 1.00 [0, 24.0] |
| Missing | 22 (0.1%) | 11 (0.0%) | 33 (0.1%) |
| **employment_status_imp** |  |  |  |
| Not employed | 8704 (33.8%) | 10933 (42.7%) | 19637 (38.2%) |
| Training/Shelter | 835 (3.2%) | 659 (2.6%) | 1494 (2.9%) |
| Part-Time/Short-Time | 2206 (8.6%) | 7525 (29.4%) | 9731 (18.9%) |
| Full-Time | 13987 (54.3%) | 6505 (25.4%) | 20492 (39.9%) |
| Missing | 9 (0.0%) | 7 (0.0%) | 16 (0.0%) |
| **work_time** |  |  |  |
| Mean (SD) | 39.8 (13.3) | 30.5 (13.7) | 35.4 (14.3) |
| Median [Min, Max] | 40.0 [0.400, 80.0] | 33.0 [0.500, 80.0] | 40.0 [0.400, 80.0] |
| Missing | 5354 (20.8%) | 7225 (28.2%) | 12579 (24.5%) |
| **current_monthly_gross_labor_income** |  |  |  |
| Mean (SD) | 3700 (19400) | 2090 (1940) | 2940 (14200) |
| Median [Min, Max] | 2800 [0, 2700000] | 1700 [0, 75600] | 2300 [0, 2700000] |
| Missing | 4968 (19.3%) | 6907 (27.0%) | 11875 (23.1%) |
| **east_german_residence** |  |  |  |
| No | 20570 (79.9%) | 20547 (80.2%) | 41117 (80.0%) |
| Yes | 5171 (20.1%) | 5082 (19.8%) | 10253 (20.0%) |
| **immigration_history** |  |  |  |
| None | 18572 (72.1%) | 18650 (72.8%) | 37222 (72.5%) |
| Indirect | 1632 (6.3%) | 1599 (6.2%) | 3231 (6.3%) |
| Direct | 5537 (21.5%) | 5380 (21.0%) | 10917 (21.3%) |
| **age_group_at_immigration** |  |  |  |
| < 6 \| Born in Germany | 20540 (79.8%) | 20607 (80.4%) | 41147 (80.1%) |
| < 12 | 381 (1.5%) | 370 (1.4%) | 751 (1.5%) |
| < 18 | 599 (2.3%) | 538 (2.1%) | 1137 (2.2%) |
| Adult | 4221 (16.4%) | 4114 (16.1%) | 8335 (16.2%) |
| **refugee_exp** |  |  |  |
| None | 23657 (91.9%) | 24213 (94.5%) | 47870 (93.2%) |
| Indirect | 76 (0.3%) | 67 (0.3%) | 143 (0.3%) |
| Direct | 1866 (7.2%) | 1217 (4.7%) | 3083 (6.0%) |
| Missing | 142 (0.6%) | 132 (0.5%) | 274 (0.5%) |
| **weight_factor** |  |  |  |
| Mean (SD) | 3100 (3990) | 3030 (3790) | 3070 (3890) |
| Median [Min, Max] | 1740 [1.24, 54200] | 1710 [1.70, 50500] | 1720 [1.24, 54200] |
| Missing | 1250 (4.9%) | 1301 (5.1%) | 2551 (5.0%) |
| **sex_entry_change** |  |  |  |
| No | 7923 (30.8%) | 8286 (32.3%) | 16209 (31.6%) |
| Yes | 95 (0.4%) | 134 (0.5%) | 229 (0.4%) |
| Missing | 17723 (68.9%) | 17209 (67.1%) | 34932 (68.0%) |
| **felt_discriminated_gender_12mo** |  |  |  |
| No | 354 (1.4%) | 372 (1.5%) | 726 (1.4%) |
| Yes | 13 (0.1%) | 129 (0.5%) | 142 (0.3%) |
| Missing | 25374 (98.6%) | 25128 (98.0%) | 50502 (98.3%) |
| **felt_discriminated_ethnic_12mo** |  |  |  |
| No | 169 (0.7%) | 316 (1.2%) | 485 (0.9%) |
| Yes | 203 (0.8%) | 183 (0.7%) | 386 (0.8%) |
| Missing | 25369 (98.6%) | 25130 (98.1%) | 50499 (98.3%) |
| **using_period_of_care** |  |  |  |
| No | 23110 (89.8%) | 22847 (89.1%) | 45957 (89.5%) |
| Yes | 94 (0.4%) | 221 (0.9%) | 315 (0.6%) |
| Missing | 2537 (9.9%) | 2561 (10.0%) | 5098 (9.9%) |
| **current_mat_parent_leave** |  |  |  |
| No | 25627 (99.6%) | 24633 (96.1%) | 50260 (97.8%) |
| Yes | 97 (0.4%) | 989 (3.9%) | 1086 (2.1%) |
| Missing | 17 (0.1%) | 7 (0.0%) | 24 (0.0%) |
| **risk_taking_scale** |  |  |  |
| Mean (SD) | 5.23 (2.45) | 4.40 (2.46) | 4.82 (2.49) |
| Median [Min, Max] | 5.00 [0, 10.0] | 5.00 [0, 10.0] | 5.00 [0, 10.0] |
| Missing | 12 (0.0%) | 16 (0.1%) | 28 (0.1%) |
| **risk_taking_driving_scale** |  |  |  |
| Mean (SD) | 3.83 (2.63) | 2.83 (2.56) | 3.32 (2.64) |
| Median [Min, Max] | 4.00 [0, 10.0] | 2.00 [0, 10.0] | 3.00 [0, 10.0] |
| Missing | 10546 (41.0%) | 9936 (38.8%) | 20482 (39.9%) |
| **risk_taking_health_scale** |  |  |  |
| Mean (SD) | 3.41 (2.49) | 2.75 (2.40) | 3.07 (2.47) |
| Median [Min, Max] | 3.00 [0, 10.0] | 2.00 [0, 10.0] | 3.00 [0, 10.0] |
| Missing | 10051 (39.0%) | 8721 (34.0%) | 18772 (36.5%) |
| **leadership_position** |  |  |  |
| No | 11079 (43.0%) | 12789 (49.9%) | 23868 (46.5%) |
| Yes | 6592 (25.6%) | 3499 (13.7%) | 10091 (19.6%) |
| Missing | 8070 (31.4%) | 9341 (36.4%) | 17411 (33.9%) |
| **frequency_homeoffice** |  |  |  |
| Never | 15824 (61.5%) | 17715 (69.1%) | 33539 (65.3%) |
| Rarely | 1770 (6.9%) | 1101 (4.3%) | 2871 (5.6%) |
| Regularly | 2149 (8.3%) | 1699 (6.6%) | 3848 (7.5%) |
| Missing | 5998 (23.3%) | 5114 (20.0%) | 11112 (21.6%) |
| **working_overtime** |  |  |  |
| No | 13258 (51.5%) | 16012 (62.5%) | 29270 (57.0%) |
| Yes | 9945 (38.6%) | 8387 (32.7%) | 18332 (35.7%) |
| Self-Employed | 2498 (9.7%) | 1205 (4.7%) | 3703 (7.2%) |
| Missing | 40 (0.2%) | 25 (0.1%) | 65 (0.1%) |
| **worried_health** |  |  |  |
| Yes | 4425 (17.2%) | 5019 (19.6%) | 9444 (18.4%) |
| Somewhat | 12543 (48.7%) | 13029 (50.8%) | 25572 (49.8%) |
| No | 8716 (33.9%) | 7534 (29.4%) | 16250 (31.6%) |
| Missing | 57 (0.2%) | 47 (0.2%) | 104 (0.2%) |
| **worried_pension** |  |  |  |
| Yes | 3770 (14.6%) | 4724 (18.4%) | 8494 (16.5%) |
| Somewhat | 7440 (28.9%) | 8074 (31.5%) | 15514 (30.2%) |
| No | 7022 (27.3%) | 5691 (22.2%) | 12713 (24.7%) |
| Missing | 7509 (29.2%) | 7140 (27.9%) | 14649 (28.5%) |
| **career_sacrifices** |  |  |  |
| No | 3926 (15.3%) | 4212 (16.4%) | 8138 (15.8%) |
| Rather no | 4799 (18.6%) | 4171 (16.3%) | 8970 (17.5%) |
| Rather yes | 3827 (14.9%) | 3372 (13.2%) | 7199 (14.0%) |
| Yes | 1355 (5.3%) | 1374 (5.4%) | 2729 (5.3%) |
| Missing | 11834 (46.0%) | 12500 (48.8%) | 24334 (47.4%) |
| **current_life_satisfaction_scale** |  |  |  |
| Mean (SD) | 7.30 (1.75) | 7.33 (1.77) | 7.32 (1.76) |
| Median [Min, Max] | 8.00 [0, 10.0] | 8.00 [0, 10.0] | 8.00 [0, 10.0] |
| Missing | 234 (0.9%) | 240 (0.9%) | 474 (0.9%) |
| **political_interest** |  |  |  |
| Not at all | 3654 (14.2%) | 5245 (20.5%) | 8899 (17.3%) |
| Not strong | 9586 (37.2%) | 12461 (48.6%) | 22047 (42.9%) |
| Strong | 9090 (35.3%) | 6530 (25.5%) | 15620 (30.4%) |
| Very strong | 3382 (13.1%) | 1375 (5.4%) | 4757 (9.3%) |
| Missing | 29 (0.1%) | 18 (0.1%) | 47 (0.1%) |
| **current_health** |  |  |  |
| Very good | 3432 (13.3%) | 2831 (11.0%) | 6263 (12.2%) |
| Good, Satisfactory | 10350 (40.2%) | 9830 (38.4%) | 20180 (39.3%) |
| Less good | 7639 (29.7%) | 8005 (31.2%) | 15644 (30.5%) |
| Bad | 3222 (12.5%) | 3805 (14.8%) | 7027 (13.7%) |
| Missing | 1098 (4.3%) | 1158 (4.5%) | 2256 (4.4%) |
| **self_esteem** |  |  |  |
| Mean (SD) | 5.78 (1.20) | 5.56 (1.34) | 5.67 (1.28) |
| Median [Min, Max] | 6.00 [1.00, 7.00] | 6.00 [1.00, 7.00] | 6.00 [1.00, 7.00] |
| Missing | 4955 (19.2%) | 4630 (18.1%) | 9585 (18.7%) |
| **health_insurance_status** |  |  |  |
| Pflichtversicherung | 12519 (48.6%) | 12874 (50.2%) | 25393 (49.4%) |
| Freiwillige Versichung | 3010 (11.7%) | 1612 (6.3%) | 4622 (9.0%) |
| Familienversicherung | 1672 (6.5%) | 3936 (15.4%) | 5608 (10.9%) |
| Rentnerversicherung | 4421 (17.2%) | 4679 (18.3%) | 9100 (17.7%) |
| Missing | 4119 (16.0%) | 2528 (9.9%) | 6647 (12.9%) |
| **alcohol_consumption** |  |  |  |
| Rarely | 5833 (22.7%) | 8598 (33.5%) | 14431 (28.1%) |
| Regularly | 6747 (26.2%) | 5876 (22.9%) | 12623 (24.6%) |
| (Almost) Daily | 2981 (11.6%) | 1271 (5.0%) | 4252 (8.3%) |
| Missing | 10180 (39.5%) | 9884 (38.6%) | 20064 (39.1%) |
| **num_physician_visits** |  |  |  |
| Mean (SD) | 2.93 (3.96) | 3.10 (3.69) | 3.02 (3.82) |
| Median [Min, Max] | 2.00 [0, 90.0] | 2.00 [0, 96.0] | 2.00 [0, 96.0] |
| Missing | 2915 (11.3%) | 1728 (6.7%) | 4643 (9.0%) |
| **highest_educational_degree** |  |  |  |
| Mean (SD) | 3.90 (1.75) | 3.68 (1.66) | 3.79 (1.71) |
| Median [Min, Max] | 3.00 [0, 8.00] | 3.00 [0, 8.00] | 3.00 [0, 8.00] |
| Missing | 1429 (5.6%) | 1393 (5.4%) | 2822 (5.5%) |
| **smoke_before_stroke** |  |  |  |
| No | 14063 (54.6%) | 16903 (66.0%) | 30966 (60.3%) |
| Yes | 11678 (45.4%) | 8726 (34.0%) | 20404 (39.7%) |
| **smoke_before_migraine** |  |  |  |
| No | 14076 (54.7%) | 16943 (66.1%) | 31019 (60.4%) |
| Yes | 11665 (45.3%) | 8686 (33.9%) | 20351 (39.6%) |
| **diabetes_before_stroke** |  |  |  |
| No | 23440 (91.1%) | 23691 (92.4%) | 47131 (91.7%) |
| Yes | 2301 (8.9%) | 1938 (7.6%) | 4239 (8.3%) |
| **diabetes_before_migraine** |  |  |  |
| No | 23430 (91.0%) | 23732 (92.6%) | 47162 (91.8%) |
| Yes | 2311 (9.0%) | 1897 (7.4%) | 4208 (8.2%) |
| **hypertension_before_stroke** |  |  |  |
| No | 18443 (71.6%) | 18996 (74.1%) | 37439 (72.9%) |
| Yes | 7298 (28.4%) | 6633 (25.9%) | 13931 (27.1%) |
| **hypertension_before_migraine** |  |  |  |
| No | 18449 (71.7%) | 19172 (74.8%) | 37621 (73.2%) |
| Yes | 7292 (28.3%) | 6457 (25.2%) | 13749 (26.8%) |
| **migraine_incidence** |  |  |  |
| No | 25218 (98.0%) | 24036 (93.8%) | 49254 (95.9%) |
| Yes | 523 (2.0%) | 1593 (6.2%) | 2116 (4.1%) |
| **stroke_incidence** |  |  |  |
| No | 25303 (98.3%) | 25325 (98.8%) | 50628 (98.6%) |
| Yes | 438 (1.7%) | 304 (1.2%) | 742 (1.4%) |

## 
