# Supplementary material for: Sex and gender effects on incidence of migraine and stroke: a longitudinal observational study based on the german socio-economic panel
Source: Biol Sex Differ. 2026 Mar 16;17:73. doi: 10.1186/s13293-026-00875-z (PMC13064216; doi:10.1186/s13293-026-00875-z)
Supplement: Supplementary file 3 — Supplementary Material 3 [file 13293_2026_875_MOESM3_ESM.docx]

## SEM (main model, unweighted)

lavaan.mi object fit to 20 imputed data sets using:
 - lavaan (0.6-21)
 - lavaan.mi (0.1-0)
See class?lavaan.mi help page for available methods.

Convergence information:
The model converged on 20 imputed data sets.
Standard errors were available for all imputations.

 Estimator ML
 Optimization method NLMINB
 Number of model parameters 50

 Number of observations 51370

Model Test User Model:

 Test statistic 55865.289
 Degrees of freedom 184
 P-value 0.000
 Pooling method D4

Model Test Baseline Model:

 Test statistic 91356.665
 Degrees of freedom 222
 P-value 0.000

User Model versus Baseline Model:

 Comparative Fit Index (CFI) 0.389
 Tucker-Lewis Index (TLI) 0.263

Loglikelihood and Information Criteria:

 Loglikelihood user model (H0) -852337.912
 Loglikelihood unrestricted model (H1) -818361.904

 Akaike (AIC) 1704775.823
 Bayesian (BIC) 1705218.164
 Sample-size adjusted Bayesian (SABIC) 1705059.263

Root Mean Square Error of Approximation:

 RMSEA 0.077
 90 Percent confidence interval - lower 0.076
 90 Percent confidence interval - upper 0.077
 P-value H_0: RMSEA <= 0.050 0.000
 P-value H_0: RMSEA >= 0.080 0.000

Standardized Root Mean Square Residual:

 SRMR 0.065

Parameter Estimates:

 Standard errors Standard
 Information Expected
 Information saturated (h1) model Structured

 Pooled across imputations Rubin's (1987) rules
 Augment within-imputation variance Scale by average RIV
 Wald test for pooled parameters t(df) distribution

 Pooled t statistics with df >= 1000 are displayed with
 df = Inf(inity) to save space. Although the t distribution
 with large df closely approximates a standard normal
 distribution, exact df for reporting these t tests can be
 obtained from parameterEstimates.mi()


Latent Variables:
 Estimate Std.Err t-value df P(>|t|) ci.lower
 gender =~
 dly_hrs_hswrk_ 0.670 0.025 26.458 32.381 0.000 0.619
 dly_hrs_chldc_ 0.804 0.042 19.086 38.510 0.000 0.719
 crrnt_mnthl___ -0.708 0.024 -29.130 Inf 0.000 -0.756
 gross_horly_wg -0.921 0.048 -19.103 24.996 0.000 -1.021
 emplymnt_stts_ -0.630 0.024 -26.257 116.012 0.000 -0.677
 hghst_dctnl_dg -0.601 0.027 -21.999 Inf 0.000 -0.655
 risk_takng_scl -0.587 0.036 -16.178 221.234 0.000 -0.659
 politicl_ntrst -0.232 0.013 -17.950 376.387 0.000 -0.258
 crrnt_mt_prnt_ 0.027 0.002 13.169 50.517 0.000 0.023
 nm_physcn_vsts 0.377 0.051 7.456 Inf 0.000 0.278
 ci.upper

 0.722
 0.890
 -0.661
 -0.822
 -0.582
 -0.548
 -0.516
 -0.207
 0.031
 0.476

Regressions:
 Estimate Std.Err t-value df
 gender ~
 sex_binary 1.000
 daily_hours_housework_weekdays ~
 nm_chldrn_n_hs 0.165 0.021 7.904 Inf
 partner 0.368 0.032 11.425 Inf
 daily_hours_childcare_weekdays ~
 nm_chldrn_n_hs 2.174 0.043 50.016 Inf
 partner 0.333 0.067 4.948 Inf
 current_monthly_gross_labor_income ~
 est_grmn_rsdnc -0.191 0.033 -5.815 Inf
 age_10y 0.084 0.007 11.841 143.256
 gross_hourly_wage ~
 est_grmn_rsdnc -0.400 0.093 -4.316 605.426
 age_10y 0.218 0.020 10.870 129.273
 migraine_incidence ~
 sex_binary 0.044 0.006 7.734 Inf
 gender -0.002 0.003 -0.477 Inf
 sex_or 0.010 0.014 0.695 Inf
 partner 0.001 0.005 0.290 Inf
 age_10y 0.004 0.002 2.534 Inf
 immigrtn_hstry -0.004 0.003 -1.339 Inf
 smoke_bfr_mgrn 0.015 0.005 3.159 Inf
 dibts_bfr_mgrn -0.012 0.009 -1.312 Inf
 hyprtnsn_bfr_m -0.016 0.006 -2.625 Inf
 stroke_incidence ~
 sex_binary -0.010 0.003 -2.997 Inf
 gender 0.006 0.002 2.951 Inf
 sex_or 0.002 0.008 0.201 Inf
 partner -0.008 0.003 -2.575 Inf
 age_10y 0.009 0.001 10.089 Inf
 immigrtn_hstry -0.001 0.002 -0.780 Inf
 smoke_bfr_strk 0.007 0.003 2.543 Inf
 dibts_bfr_strk 0.008 0.005 1.429 Inf
 hyprtnsn_bfr_s -0.001 0.004 -0.239 Inf
 P(>|t|) ci.lower ci.upper

 1.000 1.000

 0.000 0.124 0.206
 0.000 0.305 0.431

 0.000 2.089 2.259
 0.000 0.201 0.465

 0.000 -0.255 -0.126
 0.000 0.070 0.098

 0.000 -0.582 -0.218
 0.000 0.178 0.257

 0.000 0.033 0.056
 0.634 -0.008 0.005
 0.487 -0.018 0.037
 0.772 -0.008 0.011
 0.011 0.001 0.007
 0.181 -0.010 0.002
 0.002 0.006 0.025
 0.189 -0.030 0.006
 0.009 -0.029 -0.004

 0.003 -0.017 -0.004
 0.003 0.002 0.009
 0.841 -0.015 0.018
 0.010 -0.013 -0.002
 0.000 0.007 0.011
 0.435 -0.005 0.002
 0.011 0.002 0.013
 0.153 -0.003 0.019
 0.811 -0.008 0.006

Covariances:
 Estimate Std.Err t-value df P(>|t|) ci.lower
 .migraine_incidence ~~
 .stroke_incidnc 0.001 0.000 3.262 Inf 0.001 0.000
 ci.upper

 0.001

Variances:
 Estimate Std.Err t-value df P(>|t|) ci.lower
 .dly_hrs_hswrk_ 1.452 0.030 48.975 46.602 0.000 1.392
 .dly_hrs_chldc_ 6.906 0.124 55.876 211.775 0.000 6.662
 .crrnt_mnthl___ 0.944 0.023 41.902 21.243 0.000 0.897
 .gross_horly_wg 9.032 0.163 55.583 19.149 0.000 8.692
 .emplymnt_stts_ 1.332 0.027 49.360 Inf 0.000 1.279
 .hghst_dctnl_dg 2.474 0.046 54.215 278.316 0.000 2.384
 .risk_takng_scl 5.784 0.102 56.949 Inf 0.000 5.585
 .politicl_ntrst 0.685 0.012 56.351 Inf 0.000 0.661
 .crrnt_mt_prnt_ 0.020 0.000 57.690 Inf 0.000 0.019
 .nm_physcn_vsts 13.759 0.235 58.492 109.051 0.000 13.293
 .migraine_ncdnc 0.039 0.001 58.813 Inf 0.000 0.038
 .stroke_incidnc 0.014 0.000 58.735 Inf 0.000 0.013
 .gender 0.934 0.062 15.112 54.490 0.000 0.810
 ci.upper
 1.512
 7.149
 0.991
 9.372
 1.385
 2.564
 5.984
 0.709
 0.021
 14.225
 0.040
 0.014
 1.058
