# Supplementary material for: Sex and gender effects on incidence of migraine and stroke: a longitudinal observational study based on the german socio-economic panel
Source: Biol Sex Differ. 2026 Mar 16;17:73. doi: 10.1186/s13293-026-00875-z (PMC13064216; doi:10.1186/s13293-026-00875-z)
Supplement: Supplementary file 4 — Supplementary Material 4 [file 13293_2026_875_MOESM4_ESM.docx]

## Table S2: Estimates from SEM regressions by age group, unweighted

| Outcome | Predictor | [65,Inf) | [50,65) | [35,50) | [18,35) |
| --- | --- | --- | --- | --- | --- |
| migraine_incidence | sex_binary | 0.052 | 0.046 | 0.044 | 0.025 |
| migraine_incidence | gender | -0.001 | -0.004 | 0.005 | 0.001 |
| migraine_incidence | sex_or | 0.010 | 0.007 | -0.001 | 0.016 |
| migraine_incidence | partner | 0.003 | -0.006 | -0.004 | 0.010 |
| migraine_incidence | immigration_history | 0.004 | -0.007 | 0.002 | -0.013 |
| migraine_incidence | smoke_before_migraine | 0.018 | 0.021 | 0.010 | 0.009 |
| migraine_incidence | diabetes_before_migraine | -0.009 | -0.017 | -0.010 | -0.015 |
| migraine_incidence | hypertension_before_migraine | -0.015 | -0.005 | -0.022 | -0.001 |
| stroke_incidence | sex_binary | -0.023 | -0.002 | -0.010 | 0.000 |
| stroke_incidence | gender | 0.010 | 0.001 | 0.005 | 0.000 |
| stroke_incidence | sex_or | 0.001 | -0.004 | 0.007 | -0.001 |
| stroke_incidence | partner | -0.015 | -0.002 | -0.001 | 0.000 |
| stroke_incidence | immigration_history | -0.004 | -0.002 | -0.001 | 0.000 |
| stroke_incidence | smoke_before_stroke | 0.023 | 0.002 | 0.008 | 0.001 |
| stroke_incidence | diabetes_before_stroke | 0.006 | 0.006 | 0.009 | -0.001 |
| stroke_incidence | hypertension_before_stroke | -0.002 | 0.002 | 0.003 | 0.001 |
