# Supplementary material for: Sex and gender effects on incidence of migraine and stroke: a longitudinal observational study based on the german socio-economic panel
Source: Biol Sex Differ. 2026 Mar 16;17:73. doi: 10.1186/s13293-026-00875-z (PMC13064216; doi:10.1186/s13293-026-00875-z)
Supplement: Supplementary file 6 — Supplementary Material 6 [file 13293_2026_875_MOESM6_ESM.docx]

## Figure S3: Gender distributions stratified by ‘sex’ and ‘immigration history’
