# Supplementary material for: Sex and gender effects on incidence of migraine and stroke: a longitudinal observational study based on the german socio-economic panel
Source: Biol Sex Differ. 2026 Mar 16;17:73. doi: 10.1186/s13293-026-00875-z (PMC13064216; doi:10.1186/s13293-026-00875-z)
Supplement: Supplementary file 8 — Supplementary Material 8 [file 13293_2026_875_MOESM8_ESM.docx]

## Causal Discovery

We selected an expanded set of potentially gender-related variables based on previous research and applied a causal discovery technique aiming to uncover additional causal relationships from the data.

## Causal Discovery: Continuous Imputed Data

dag {
age
age_group_at_immigration
alcohol_consumption
career_sacrifices
current_health
current_life_satisfaction_scale
current_mat_parent_leave
current_monthly_gross_labor_income
daily_hours_childcare_weekdays
daily_hours_housework_weekdays
diabetes
diabetes_before_migraine
diabetes_before_stroke
east_german_residence
employment_status_imp
felt_discriminated_ethnic_12mo
felt_discriminated_gender_12mo
frequency_homeoffice
gender
gross_hourly_wage
health_insurance_status
highest_educational_degree
hypertension
hypertension_before_migraine
hypertension_before_stroke
immigration_history
leadership_position
migraine
migraine_incidence
num_children_in_household
num_physician_visits
partner
political_interest
refugee_exp
risk_taking_driving_scale
risk_taking_health_scale
risk_taking_scale
self_esteem
sex_binary
sex_entry_change
sex_or
smoke
smoke_before_migraine
smoke_before_stroke
stroke
stroke_incidence
using_period_of_care
work_time
working_overtime
worried_health
worried_pension
age -> gross_hourly_wage
age -> hypertension_before_migraine
age -> hypertension_before_stroke
age -> num_children_in_household
age -> stroke_incidence
age_group_at_immigration -> hypertension_before_migraine
alcohol_consumption -> age
alcohol_consumption -> current_monthly_gross_labor_income
alcohol_consumption -> diabetes_before_stroke
alcohol_consumption -> highest_educational_degree
alcohol_consumption -> migraine
alcohol_consumption -> smoke_before_stroke
career_sacrifices -> age
career_sacrifices -> current_health
career_sacrifices -> current_life_satisfaction_scale
career_sacrifices -> highest_educational_degree
career_sacrifices -> migraine_incidence
career_sacrifices -> work_time
current_health -> age
current_health -> employment_status_imp
current_health -> migraine_incidence
current_health -> num_physician_visits
current_health -> self_esteem
current_health -> stroke_incidence
current_health -> worried_health
current_life_satisfaction_scale -> current_health
current_life_satisfaction_scale -> current_monthly_gross_labor_income
current_life_satisfaction_scale -> num_physician_visits
current_life_satisfaction_scale -> self_esteem
current_mat_parent_leave -> age
current_mat_parent_leave -> current_life_satisfaction_scale
current_mat_parent_leave -> gender
current_mat_parent_leave -> working_overtime
current_monthly_gross_labor_income -> gender
current_monthly_gross_labor_income -> work_time
daily_hours_childcare_weekdays -> current_mat_parent_leave
daily_hours_childcare_weekdays -> gender
daily_hours_childcare_weekdays -> num_children_in_household
daily_hours_childcare_weekdays -> political_interest
daily_hours_housework_weekdays -> current_monthly_gross_labor_income
daily_hours_housework_weekdays -> gender
daily_hours_housework_weekdays -> work_time
diabetes -> diabetes_before_migraine
diabetes -> diabetes_before_stroke
diabetes -> hypertension
diabetes_before_migraine -> hypertension_before_migraine
diabetes_before_migraine -> migraine_incidence
diabetes_before_migraine -> stroke_incidence
diabetes_before_stroke -> diabetes_before_migraine
diabetes_before_stroke -> hypertension_before_stroke
east_german_residence -> age_group_at_immigration
east_german_residence -> alcohol_consumption
east_german_residence -> career_sacrifices
east_german_residence -> current_life_satisfaction_scale
east_german_residence -> current_monthly_gross_labor_income
east_german_residence -> felt_discriminated_ethnic_12mo
east_german_residence -> gross_hourly_wage
east_german_residence -> highest_educational_degree
east_german_residence -> hypertension_before_migraine
east_german_residence -> migraine
east_german_residence -> num_physician_visits
east_german_residence -> sex_entry_change
east_german_residence -> work_time
east_german_residence -> worried_health
east_german_residence -> worried_pension
employment_status_imp -> age
employment_status_imp -> gender
employment_status_imp -> smoke
employment_status_imp -> stroke_incidence
employment_status_imp -> work_time
felt_discriminated_ethnic_12mo -> age_group_at_immigration
felt_discriminated_ethnic_12mo -> alcohol_consumption
felt_discriminated_ethnic_12mo -> diabetes_before_migraine
felt_discriminated_ethnic_12mo -> highest_educational_degree
felt_discriminated_ethnic_12mo -> migraine
felt_discriminated_ethnic_12mo -> migraine_incidence
felt_discriminated_ethnic_12mo -> self_esteem
felt_discriminated_ethnic_12mo -> stroke
felt_discriminated_ethnic_12mo -> using_period_of_care
felt_discriminated_ethnic_12mo -> worried_health
felt_discriminated_gender_12mo -> age
felt_discriminated_gender_12mo -> age_group_at_immigration
felt_discriminated_gender_12mo -> career_sacrifices
felt_discriminated_gender_12mo -> current_health
felt_discriminated_gender_12mo -> felt_discriminated_ethnic_12mo
felt_discriminated_gender_12mo -> hypertension_before_migraine
felt_discriminated_gender_12mo -> hypertension_before_stroke
felt_discriminated_gender_12mo -> migraine
felt_discriminated_gender_12mo -> political_interest
felt_discriminated_gender_12mo -> risk_taking_health_scale
felt_discriminated_gender_12mo -> self_esteem
felt_discriminated_gender_12mo -> stroke
felt_discriminated_gender_12mo -> worried_health
frequency_homeoffice -> alcohol_consumption
frequency_homeoffice -> career_sacrifices
frequency_homeoffice -> current_monthly_gross_labor_income
frequency_homeoffice -> employment_status_imp
frequency_homeoffice -> gross_hourly_wage
frequency_homeoffice -> health_insurance_status
frequency_homeoffice -> highest_educational_degree
frequency_homeoffice -> num_children_in_household
frequency_homeoffice -> political_interest
frequency_homeoffice -> smoke_before_migraine
frequency_homeoffice -> working_overtime
gross_hourly_wage -> current_monthly_gross_labor_income
gross_hourly_wage -> gender
health_insurance_status -> age
health_insurance_status -> employment_status_imp
health_insurance_status -> hypertension_before_migraine
health_insurance_status -> smoke
health_insurance_status -> work_time
highest_educational_degree -> current_monthly_gross_labor_income
highest_educational_degree -> gender
highest_educational_degree -> gross_hourly_wage
highest_educational_degree -> smoke
hypertension -> hypertension_before_migraine
hypertension -> hypertension_before_stroke
hypertension -> migraine_incidence
hypertension -> num_children_in_household
hypertension -> smoke
hypertension_before_migraine -> stroke_incidence
hypertension_before_stroke -> hypertension_before_migraine
hypertension_before_stroke -> migraine_incidence
immigration_history -> age
immigration_history -> age_group_at_immigration
immigration_history -> alcohol_consumption
immigration_history -> career_sacrifices
immigration_history -> east_german_residence
immigration_history -> felt_discriminated_ethnic_12mo
immigration_history -> leadership_position
immigration_history -> num_children_in_household
immigration_history -> political_interest
immigration_history -> refugee_exp
immigration_history -> sex_entry_change
leadership_position -> alcohol_consumption
leadership_position -> career_sacrifices
leadership_position -> current_monthly_gross_labor_income
leadership_position -> frequency_homeoffice
leadership_position -> highest_educational_degree
leadership_position -> political_interest
leadership_position -> self_esteem
leadership_position -> work_time
leadership_position -> working_overtime
migraine -> hypertension_before_migraine
migraine -> migraine_incidence
migraine -> smoke_before_migraine
migraine -> smoke_before_stroke
migraine -> stroke_incidence
migraine_incidence -> stroke_incidence
num_children_in_household -> hypertension_before_stroke
num_children_in_household -> smoke_before_stroke
num_physician_visits -> diabetes
num_physician_visits -> employment_status_imp
num_physician_visits -> gender
num_physician_visits -> stroke
partner -> age
partner -> age_group_at_immigration
partner -> current_life_satisfaction_scale
partner -> current_monthly_gross_labor_income
partner -> daily_hours_childcare_weekdays
partner -> daily_hours_housework_weekdays
partner -> health_insurance_status
partner -> highest_educational_degree
partner -> leadership_position
partner -> num_children_in_household
partner -> smoke
partner -> working_overtime
political_interest -> age
political_interest -> alcohol_consumption
political_interest -> gender
political_interest -> highest_educational_degree
political_interest -> smoke
refugee_exp -> age_group_at_immigration
refugee_exp -> alcohol_consumption
refugee_exp -> felt_discriminated_ethnic_12mo
refugee_exp -> felt_discriminated_gender_12mo
refugee_exp -> health_insurance_status
refugee_exp -> highest_educational_degree
refugee_exp -> migraine_incidence
refugee_exp -> num_children_in_household
refugee_exp -> risk_taking_scale
refugee_exp -> self_esteem
refugee_exp -> sex_entry_change
refugee_exp -> using_period_of_care
risk_taking_driving_scale -> age
risk_taking_driving_scale -> age_group_at_immigration
risk_taking_driving_scale -> alcohol_consumption
risk_taking_driving_scale -> employment_status_imp
risk_taking_driving_scale -> leadership_position
risk_taking_driving_scale -> risk_taking_health_scale
risk_taking_driving_scale -> work_time
risk_taking_driving_scale -> working_overtime
risk_taking_health_scale -> age
risk_taking_health_scale -> alcohol_consumption
risk_taking_health_scale -> num_physician_visits
risk_taking_health_scale -> self_esteem
risk_taking_health_scale -> smoke
risk_taking_scale -> age
risk_taking_scale -> age_group_at_immigration
risk_taking_scale -> current_health
risk_taking_scale -> current_life_satisfaction_scale
risk_taking_scale -> current_monthly_gross_labor_income
risk_taking_scale -> daily_hours_housework_weekdays
risk_taking_scale -> gender
risk_taking_scale -> leadership_position
risk_taking_scale -> risk_taking_driving_scale
risk_taking_scale -> risk_taking_health_scale
risk_taking_scale -> self_esteem
risk_taking_scale -> working_overtime
self_esteem -> age_group_at_immigration
self_esteem -> migraine_incidence
sex_binary -> alcohol_consumption
sex_binary -> current_mat_parent_leave
sex_binary -> daily_hours_childcare_weekdays
sex_binary -> daily_hours_housework_weekdays
sex_binary -> diabetes
sex_binary -> felt_discriminated_ethnic_12mo
sex_binary -> felt_discriminated_gender_12mo
sex_binary -> gender
sex_binary -> gross_hourly_wage
sex_binary -> migraine_incidence
sex_binary -> refugee_exp
sex_binary -> risk_taking_driving_scale
sex_binary -> self_esteem
sex_binary -> stroke_incidence
sex_binary -> work_time
sex_entry_change -> alcohol_consumption
sex_entry_change -> career_sacrifices
sex_entry_change -> daily_hours_housework_weekdays
sex_entry_change -> diabetes_before_stroke
sex_entry_change -> employment_status_imp
sex_entry_change -> felt_discriminated_ethnic_12mo
sex_entry_change -> felt_discriminated_gender_12mo
sex_entry_change -> migraine
sex_entry_change -> risk_taking_scale
sex_entry_change -> smoke_before_migraine
sex_entry_change -> smoke_before_stroke
sex_or -> age
sex_or -> felt_discriminated_ethnic_12mo
sex_or -> felt_discriminated_gender_12mo
sex_or -> frequency_homeoffice
sex_or -> migraine
sex_or -> num_children_in_household
sex_or -> num_physician_visits
sex_or -> partner
sex_or -> political_interest
sex_or -> risk_taking_health_scale
sex_or -> self_esteem
sex_or -> smoke
smoke -> smoke_before_migraine
smoke -> smoke_before_stroke
smoke_before_migraine -> migraine_incidence
smoke_before_migraine -> stroke_incidence
smoke_before_stroke -> migraine_incidence
smoke_before_stroke -> smoke_before_migraine
smoke_before_stroke -> stroke_incidence
stroke -> diabetes_before_stroke
stroke -> migraine_incidence
stroke -> smoke_before_migraine
stroke -> smoke_before_stroke
stroke -> stroke_incidence
using_period_of_care -> age_group_at_immigration
using_period_of_care -> alcohol_consumption
using_period_of_care -> current_life_satisfaction_scale
using_period_of_care -> daily_hours_housework_weekdays
using_period_of_care -> employment_status_imp
using_period_of_care -> gender
using_period_of_care -> work_time
using_period_of_care -> worried_health
work_time -> gross_hourly_wage
work_time -> smoke
working_overtime -> alcohol_consumption
working_overtime -> career_sacrifices
working_overtime -> current_monthly_gross_labor_income
working_overtime -> employment_status_imp
working_overtime -> health_insurance_status
working_overtime -> stroke_incidence
working_overtime -> work_time
worried_health -> career_sacrifices
worried_health -> current_life_satisfaction_scale
worried_health -> diabetes
worried_health -> num_physician_visits
worried_health -> stroke_incidence
worried_health -> worried_pension
worried_pension -> age
worried_pension -> alcohol_consumption
worried_pension -> career_sacrifices
worried_pension -> current_life_satisfaction_scale
worried_pension -> current_monthly_gross_labor_income
worried_pension -> employment_status_imp
worried_pension -> health_insurance_status
worried_pension -> migraine
worried_pension -> self_esteem
worried_pension -> smoke
}

## Causal Discovery: Categorical Raw Data

dag {
age
age_group_at_immigration
alcohol_consumption
career_sacrifices
current_health
current_life_satisfaction_scale
current_mat_parent_leave
current_monthly_gross_labor_income
daily_hours_childcare_weekdays
daily_hours_housework_weekdays
diabetes
diabetes_before_migraine
diabetes_before_stroke
east_german_residence
employment_status_imp
felt_discriminated_ethnic_12mo
felt_discriminated_gender_12mo
frequency_homeoffice
gender
gross_hourly_wage
health_insurance_status
highest_educational_degree
hypertension
hypertension_before_migraine
hypertension_before_stroke
immigration_history
leadership_position
migraine
migraine_incidence
num_children_in_household
num_physician_visits
partner
political_interest
refugee_exp
risk_taking_driving_scale
risk_taking_health_scale
risk_taking_scale
self_esteem
sex_binary
sex_entry_change
sex_or
smoke
smoke_before_migraine
smoke_before_stroke
stroke
stroke_incidence
using_period_of_care
work_time
working_overtime
worried_health
worried_pension
age -> num_children_in_household
alcohol_consumption -> current_monthly_gross_labor_income
career_sacrifices -> work_time
current_health -> gross_hourly_wage
current_health -> self_esteem
current_life_satisfaction_scale -> current_health
current_life_satisfaction_scale -> self_esteem
current_life_satisfaction_scale -> smoke
current_monthly_gross_labor_income -> gender
current_monthly_gross_labor_income -> gross_hourly_wage
current_monthly_gross_labor_income -> hypertension_before_migraine
current_monthly_gross_labor_income -> smoke
current_monthly_gross_labor_income -> work_time
daily_hours_housework_weekdays -> current_health
daily_hours_housework_weekdays -> health_insurance_status
daily_hours_housework_weekdays -> num_children_in_household
daily_hours_housework_weekdays -> worried_pension
diabetes_before_migraine -- diabetes_before_stroke
diabetes_before_migraine -> hypertension_before_migraine
east_german_residence -> career_sacrifices
employment_status_imp -> health_insurance_status
felt_discriminated_gender_12mo -> sex_binary
frequency_homeoffice -> risk_taking_health_scale
health_insurance_status -> age
health_insurance_status -> gross_hourly_wage
health_insurance_status -> work_time
hypertension -- hypertension_before_stroke
hypertension -> hypertension_before_migraine
hypertension_before_stroke -> hypertension_before_migraine
immigration_history -> age
immigration_history -> age_group_at_immigration
leadership_position -> current_monthly_gross_labor_income
leadership_position -> work_time
migraine -- migraine_incidence
num_physician_visits -> current_health
partner -> current_monthly_gross_labor_income
political_interest -> gross_hourly_wage
political_interest -> self_esteem
refugee_exp -> age_group_at_immigration
risk_taking_driving_scale -> leadership_position
risk_taking_driving_scale -> risk_taking_health_scale
risk_taking_driving_scale -> smoke
risk_taking_health_scale -> smoke
risk_taking_health_scale -> worried_health
risk_taking_scale -> alcohol_consumption
risk_taking_scale -> current_life_satisfaction_scale
risk_taking_scale -> gender
risk_taking_scale -> political_interest
risk_taking_scale -> risk_taking_driving_scale
risk_taking_scale -> risk_taking_health_scale
risk_taking_scale -> smoke
sex_binary -> current_monthly_gross_labor_income
sex_or -> num_children_in_household
smoke_before_migraine -- smoke_before_stroke
smoke_before_stroke -> smoke
stroke -- stroke_incidence
work_time -> gender
working_overtime -> career_sacrifices
worried_health -> current_monthly_gross_labor_income
worried_health -> gross_hourly_wage
worried_pension -> current_life_satisfaction_scale
worried_pension -> worried_health
}
