# Supplementary figures and images for: Sex and gender effects on incidence of migraine and stroke: a longitudinal observational study based on the german socio-economic panel
Source: Biol Sex Differ. 2026 Mar 16;17:73. doi: 10.1186/s13293-026-00875-z (PMC13064216; doi:10.1186/s13293-026-00875-z)

## Figure S1: Pearson correlation plot for missingness


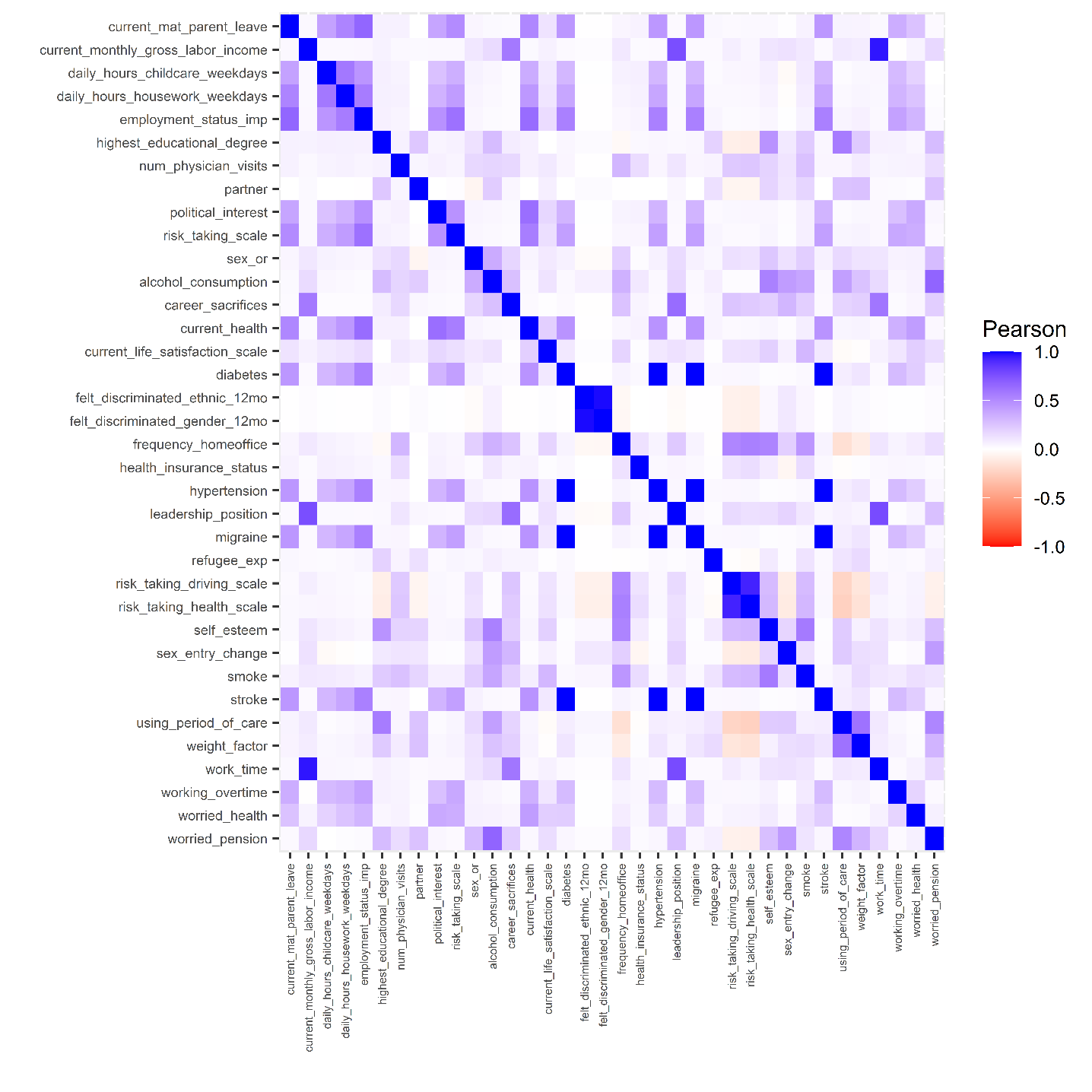

Supplement: Supplementary file 9 — Supplementary Material 9 [file 13293_2026_875_MOESM9_ESM.docx]
