# Supplementary material for: Sex and gender effects on incidence of migraine and stroke: a longitudinal observational study based on the german socio-economic panel
Source: Biol Sex Differ. 2026 Mar 16;17:73. doi: 10.1186/s13293-026-00875-z (PMC13064216; doi:10.1186/s13293-026-00875-z)
Supplement: Supplementary file 10 — Supplementary Material 10 [file 13293_2026_875_MOESM10_ESM.docx]

## SEM (main model)

# measurement model for latent variable gender
 gender =~ daily_hours_housework_weekdays + daily_hours_childcare_weekdays +
 current_monthly_gross_labor_income + gross_hourly_wage + employment_status_imp +
 highest_educational_degree + risk_taking_scale + political_interest + current_mat_parent_leave + num_physician_visits

 # hypothesis: sex at birth -> gender
 gender ~ 1.0 * sex_binary

 # regressions between other exogeneous variables and gender predictors
 # hypotheses: +children => +housework, +partner => -housework (shares) or +housework
 # hypotheses: +children => +childcare, +partner => -childcare (shares)
 # hypotheses: +east => -income, -wage, +age => +income, +wage
 daily_hours_housework_weekdays ~ num_children_in_household + partner
 daily_hours_childcare_weekdays ~ num_children_in_household + partner
 current_monthly_gross_labor_income ~ east_german_residence + age_10y
 gross_hourly_wage ~ east_german_residence + age_10y

 migraine_incidence ~ sex_binary + gender + sex_or + partner + age_10y + immigration_history +
 smoke_before_migraine + diabetes_before_migraine + hypertension_before_migraine
 stroke_incidence ~ sex_binary + gender + sex_or + partner + age_10y + immigration_history +
 smoke_before_stroke + diabetes_before_stroke + hypertension_before_stroke

## SEM (sensitivity analysis model without link between sex and gender)

# measurement model for latent variable gender
 gender =~ daily_hours_housework_weekdays + daily_hours_childcare_weekdays +
 current_monthly_gross_labor_income + gross_hourly_wage + employment_status_imp +
 highest_educational_degree + risk_taking_scale + political_interest + current_mat_parent_leave + num_physician_visits

 # fix variance for gender, comparable dynamic range to sex_binary
 gender ~~ 1.0 * gender

 # regressions between other exogeneous variables and gender predictors
 # hypotheses: +children => +housework, +partner => -housework (shares) or +housework
 # hypotheses: +children => +childcare, +partner => -childcare (shares)
 # hypotheses: +east => -income, -wage, +age => +income, +wage
 daily_hours_housework_weekdays ~ num_children_in_household + partner
 daily_hours_childcare_weekdays ~ num_children_in_household + partner
 current_monthly_gross_labor_income ~ east_german_residence + age_10y
 gross_hourly_wage ~ east_german_residence + age_10y

 migraine_incidence ~ sex_binary + gender + sex_or + partner + age_10y + immigration_history +
 smoke_before_migraine + diabetes_before_migraine + hypertension_before_migraine
 stroke_incidence ~ sex_binary + gender + sex_or + partner + age_10y + immigration_history +
 smoke_before_stroke + diabetes_before_stroke + hypertension_before_stroke
