# Supplementary material for: Sex and gender effects on incidence of migraine and stroke: a longitudinal observational study based on the german socio-economic panel
Source: Biol Sex Differ. 2026 Mar 16;17:73. doi: 10.1186/s13293-026-00875-z (PMC13064216; doi:10.1186/s13293-026-00875-z)
Supplement: Supplementary file 11 — Supplementary Material 11 [file 13293_2026_875_MOESM11_ESM.docx]

## Table S3: SEM estimates with 95% confidence intervals (unweighted, weighted, sensitivity analysis)

| Outcome | Predictor | Unweighted | | Weighted | | Sensitivity | |
| --- | --- | --- | --- | --- | --- | --- | --- |
| migraine | sex_binary | 0.044 [0.033, 0.056] | *** | 0.048 [0.039, 0.057] | *** | 0.043 [0.033, 0.053] | *** |
| migraine | gender | -0.002 [-0.008, 0.005] |  | 0.000 [-0.004, 0.004] |  | -0.001 [-0.007, 0.005] |  |
| migraine | sex_or | 0.010 [-0.018, 0.037] |  | 0.006 [-0.017, 0.030] |  | 0.010 [-0.019, 0.039] |  |
| migraine | partner | 0.001 [-0.008, 0.011] |  | 0.002 [-0.006, 0.010] |  | 0.002 [-0.009, 0.012] |  |
| migraine | age_10y | 0.004 [0.001, 0.007] | * | 0.005 [0.002, 0.007] | *** | 0.004 [0.001, 0.007] | * |
| migraine | immigration_history | -0.004 [-0.010, 0.002] |  | -0.000 [-0.006, 0.005] |  | -0.004 [-0.010, 0.002] |  |
| migraine | smoke_before_migraine | 0.015 [0.006, 0.025] | ** | 0.010 [0.002, 0.018] | * | 0.015 [0.005, 0.026] | ** |
| migraine | diabetes_before_migraine | -0.012 [-0.030, 0.006] |  | -0.013 [-0.025, -0.001] | * | -0.012 [-0.031, 0.007] |  |
| migraine | hypertension_before_migraine | -0.016 [-0.029, -0.004] | ** | -0.028 [-0.037, -0.018] | *** | -0.016 [-0.029, -0.004] | * |
| stroke | sex_binary | -0.010 [-0.017, -0.004] | ** | -0.013 [-0.018, -0.007] | *** | -0.007 [-0.013, -0.001] | * |
| stroke | gender | 0.006 [0.002, 0.009] | ** | 0.006 [0.004, 0.009] | *** | 0.005 [0.002, 0.009] | ** |
| stroke | sex_or | 0.002 [-0.015, 0.018] |  | 0.003 [-0.007, 0.013] |  | 0.002 [-0.016, 0.019] |  |
| stroke | partner | -0.008 [-0.013, -0.002] | * | -0.008 [-0.013, -0.002] | ** | -0.008 [-0.014, -0.002] | * |
| stroke | age_10y | 0.009 [0.007, 0.011] | *** | 0.012 [0.010, 0.013] | *** | 0.009 [0.007, 0.011] | *** |
| stroke | immigration_history | -0.001 [-0.005, 0.002] |  | -0.002 [-0.005, 0.002] |  | -0.001 [-0.005, 0.002] |  |
| stroke | smoke_before_stroke | 0.007 [0.002, 0.013] | * | 0.009 [0.004, 0.014] | *** | 0.007 [0.001, 0.013] | * |
| stroke | diabetes_before_stroke | 0.008 [-0.003, 0.019] |  | 0.012 [-0.001, 0.026] |  | 0.008 [-0.003, 0.019] |  |
| stroke | hypertension_before_stroke | -0.001 [-0.008, 0.006] |  | -0.003 [-0.010, 0.005] |  | -0.001 [-0.008, 0.007] |  |

## Table S4: Fit metrics (for unweighted main SEM, weighted SEM, age-group stratified SEM, and sensitivity analysis SEM)

| Metric | Unweighted | Weighted | Age group | Sensitivity |
| --- | --- | --- | --- | --- |
| RMSEA: Root Mean Square Error of Approximation | 0.077 | 0.077 | 0.058 | 0.085 |
| SRMSR: Standardized Root Mean Square Residual | 0.065 | 0.072 | 0.050 | 0.071 |
| AIC: Akaike Information Criterion | 1,704,775.823 | 1,738,766.022 | 1,540,760.800 | 1,710,988.942 |

## 
